# Decentralized queue control with delay shifting in edge-IoT using reinforcement learning

**DOI:** 10.1038/s41598-025-13983-4

**Published:** 2025-08-22

**Authors:** Viacheslav Kovtun

**Affiliations:** Vinnytsia National Technical University, Vinnytsia, Ukraine

**Keywords:** Applied mathematics, Computational science, Information technology

## Abstract

The article presents an adaptive approach to modelling and managing the service process of requests at peripheral nodes of edge-IoT systems. This approach is highly relevant in light of increasing demands for energy efficiency, responsiveness, and self-regulation under unstable traffic conditions. A stochastic G/G/1 model with a parameterised time shift is proposed, accounting for the temporary unavailability of the device prior to request processing. Analytical expressions for key QoS indicators (delay, variability, loss, energy consumption) as functions of the shift parameter are derived, and a multi-factor reward function is constructed. A DQN-based reinforcement learning agent architecture is implemented to dynamically control the shift parameter in a decentralised manner based on the local real-time queue state. Experimental results using real-world datasets demonstrated a reduction in average delay by 17–26%, decreased fluctuations in service time, and improved queue recovery stability after peak loads compared to current state-of-the-art models. The proposed solution is traffic-type agnostic and scalable across edge architectures of varying complexity. The results are suitable for deployment in sensor networks, 5G/6G edge scenarios, and systems with dynamic QoS and energy management.

## Introduction

### Relevance of the research

Edge-IoT^1–3^ represents a new evolutionary phase of the Internet of Things, which entails performing computational operations directly at the network’s edge nodes, such as sensors, gateways, and microcontrollers. This approach reduces latency, alleviates cloud load, enhances autonomy, and ensures instant response to events. It is anticipated that by 2025, the majority of IoT applications will adopt edge architecture, with the number of interconnected devices exceeding 50 billion. However, alongside infrastructure advancement, challenges related to the instability of real-time data processing are becoming increasingly pronounced.

In the deployment of edge-IoT systems, particularly in smart buildings, cities, industrial facilities, and healthcare institutions, failures are increasingly observed due to the inability of edge devices to process requests with adequate speed. For instance, in smart building monitoring systems, where thousands of sensors detect changes in temperature, humidity, smoke, or movement, an excessive accumulation of events often leads to avalanche-like processing overload. As a result, data are either delayed or lost, and alarm systems may fail to activate. On a city-wide scale, these issues become more complex. In locations such as Singapore or Barcelona, adaptive control systems for traffic, lighting, or metro ventilation rely on edge processing. However, during peak hours or emergency situations, local nodes often fail to process all incoming requests, thereby reducing response accuracy and speed. In industry, especially in the energy sector, edge analytics are employed to monitor pressure, vibrations, or leakages for early warning. Nevertheless, due to energy constraints, nodes frequently operate with delays caused by sleep modes and are not always ready to respond immediately to incoming events. Similar challenges are recorded in healthcare, particularly in wearable devices monitoring heart rate or glucose levels, where a delay in the activation of the processing module may have fatal consequences.

A common feature across all these cases is the presence of a time between the moment a request arrives and the moment the device is physically capable of processing it. This shift may be caused by the microcontroller’s wake-up phase, protocol initialisation, the need to wait for the completion of a previous cycle, or even hardware-related inertia. However, in most existing models, such delays are either ignored, treated as stochastic fluctuations, or compensated for by a hypothetical “reserve”. Standard queue management policies, as analysed in subsection 1.2, fail to account for the structured nature of such shifts and therefore cannot adequately manage buffering under high load variability. As a result, systems experience not only queue overloads, increased waiting times, and request losses, but also elevated energy consumption, inefficient use of communication channels, and failures in executing critical tasks. In edge-IoT conditions, this signifies not merely service degradation but, in many cases, a threat to the safety and resilience of the system. Despite the evident need to consider phase delays in processing operations, current queue management models do not incorporate such shifts within a formalised framework, and thus lack the means for their prediction or adaptive control.

### State-of-the-art

Despite extensive attention to the optimisation of delay and buffering in edge-IoT environments, there remains no universal solution that simultaneously considers input traffic variability, hardware-level constraints of edge nodes, and the structural phase delay occurring between request arrival and the device’s readiness for processing. Existing approaches address different facets of the problem, yet each possesses both strengths and significant limitations. To identify the scientific and practical foundations of our study, we shall examine the key paradigms for managing queues and delays in peripheral IoT systems, with a particular focus on their capacity to model or compensate for processing phase shifts.

Classical stochastic queueing models such as M/M/1, M/G/1, and G/G/1^4–6^ are widely employed to describe the arrival, processing, and accumulation of requests in a buffer. In edge-IoT systems, they serve as a foundation for the analytical evaluation of delay, loss, and node load, particularly in the context of protocols such as LoRaWAN, NB-IoT, URLLC, or 5G nodes, where they are used to establish theoretical performance bounds. Despite their mathematical formalisation, these models exhibit significant limitations in applied scenarios. The most critical of these is the assumption of the device’s instantaneous readiness for service, which fails to account for periods of unavailability due to initialisation, protocol delays, energy-saving pauses, or software processing. Furthermore, these models are static. They do not adapt to fluctuations in load or changes in device behaviour, which are typical in edge-IoT environments characterised by high traffic variability. Consequently, despite their analytical generality, traditional stochastic queues do not provide accurate modelling of service dynamics in edge-IoT, especially under structurally induced delays.

In queue management practice within IoT networks, standard algorithms such as DropTail, RED (Random Early Detection), and CoDel (Controlled Delay) are widely used. Originally developed for telecommunication systems, they have been adapted to edge-IoT as embedded policies within routers, gateways, or fog nodes. DropTail^7^ operates on the principle of a full buffer, RED^8^ employs a probabilistic early drop mechanism, and CoDel^9^ discards packets that exceed a defined queueing time. Their objective is to reduce latency and stabilise the queue without compromising QoS. Despite their widespread adoption (notably in LoRaWAN, NB-IoT, URLLC), these approaches exhibit significant limitations in heterogeneous environments. They fail to consider the internal state of a node (particularly its phase of unavailability due to initialisation or energy-saving cycles). Drop decisions are based solely on macrometrics (queue length, buffer time), without regard for the actual readiness of the device. Moreover, these policies are rigidly defined, lack self-learning capabilities, and are unable to adapt to changing traffic patterns. In unstable environments, even minor delays can critically impair service quality. None of these approaches models the node’s readiness window, resulting in a disconnect between queue logic and the actual operational state of the device.

In edge-IoT systems with constrained energy and computational resources, a substantial portion of delay is caused not only by servicing algorithms but also by protocol-dependent mechanisms. Modes such as PSM and eDRX in NB-IoT^10^, duty cycle restrictions in LoRaWAN^11^, and MAC-level regulations in BLE, ZigBee, and 6LoWPAN^12^ define periods of device unavailability that are regular and predictable, yet uncontrollable from the application logic level. These mechanisms operate through timers, wake-up policies, and service intervals embedded in the protocol stack, without provision for adaptation to queue state or event priority. Under peak load or URLLC requirements, such inflexibility results in event loss or unacceptable delays. Their configuration requires firmware-level modifications, which limits adaptability. Since the queue lacks access to the protocol’s phase structure, classical models are incapable of accurately predicting system behaviour.

In the deployment of edge-IoT systems, heuristic queue management strategies^13–16^ are frequently employed. These are based on a priori knowledge or empirical patterns rather than formal stochastic models. They typically involve fixed or conditional rules, such as dropping requests once a queue threshold is exceeded or delaying processing to reduce initial load. Such approaches are common in smart lighting systems and sensor networks, where logic is confined to the local context. Despite their simplicity, intuitiveness, and low computational overhead, heuristics exhibit critical limitations: rigid thresholding, lack of adaptation to traffic variation, and inconsistency with the current state of the queue or node. Their most significant drawback is the disregard for the device’s phase of unavailability. A predefined shift or delay remains unchanged regardless of the system’s resource state, which, under dynamic load conditions, leads to degraded service quality. Due to the absence of a formalised model, such strategies cannot ensure stable control or maintain efficiency in variable environments.

In response to the limited resources of edge nodes and unstable traffic loads, offloading approaches are actively evolving, aiming to delegate processing to other nodes or the cloud. This facilitates load balancing through peer-to-peer interaction or request redirection to the fog layer, as implemented in Cloudlets^16^, EdgeX Foundry^17^, and OpenFog^18^. Algorithms range from static to dynamic, with some incorporating learning based on historical data. While effective in distributing workload, these systems do not model the internal delay at the receiving node. It is typically assumed that processing begins immediately upon transmission. This overlooks activation phases, initialisation, or protocol constraints, thereby distorting the actual queue dynamics. Offloading also introduces additional network delay, which is critical in URLLC or medical systems where the precision of processing start time is vital. Moreover, decisions are based on external metrics, without accounting for the temporal readiness of the target node, which may lead to request transmission at a moment when the node is unavailable.

With the increasing computational capacity at the edge and the growing volume of telemetry data, there is a rising interest in applying reinforcement learning (RL) for optimising queue management in edge-IoT systems^19–21^. Unlike classical or heuristic approaches, RL enables the construction of policies based on empirical regularities. An agent learns through interaction with the environment, making decisions that maximise a target function, typically defined in terms of QoS indicators such as delay, loss, or energy consumption. The literature reports applications of Q-learning, DQN, actor-critic, and policy gradient methods for tuning RED, managing buffers, and implementing offloading strategies. What follows is an analysis of RL-based delay management methods in Edge-IoT that are semantically closest to the present study.

Article^22^ presents a task scheduling model in edge-IoT environments based on the DQN algorithm. The problem is formulated as a Markov decision process in which the agent learns to allocate tasks across virtual machines of the edge server, considering their deadlines, arrival times, and resource constraints. The model demonstrates the ability to reduce the number of overdue tasks and improve the on-time completion rate. However, the proposed approach focuses solely on external task assignment and does not account for the internal structure of the service process: queueing mechanisms, device phase unavailability, and parameterised delay control are excluded from the analytical model. Article^23^ concentrates on applying deep reinforcement learning (DRL) for adaptive computational load distribution among edge nodes, aiming to reduce task execution time and failure rates. The model makes decisions based on current node load, task complexity, and network delay, implementing an external balancing mechanism without a formal description of the internal service structure. The queue is not modelled as a component of a service system, while phase unavailability, structural delay, and traffic variability remain outside the analytical framework. Article^24^ introduces the DRL-based SEE-MTS model for safe and energy-efficient task allocation in edge environments, considering task class, overload risk, and energy consumption. Despite its multifactor nature, the model operates only at a high-level scheduling layer, without accounting for internal queue dynamics, phase unavailability, or parameterised delay control. Article^25^ addresses the problem of distributing computational and network resources in fog-IoT networks under delay constraints. The authors formalise the task as a stochastic optimisation problem and apply a reinforcement learning model to synthesise a control policy under partial system state information. Although delay is integrated as a target metric, it is treated as an aggregate value without distinguishing its structural components: the queue is not formalised, and node phase inertia and parameter-controlled service shifts are not incorporated. Article^26^ proposes a DRL approach to active queue management (AQM) in IoT networks, aiming to minimise average delay, service time variation (jitter), and packet loss. The authors employ DQN to train an agent that dynamically adjusts the service policy based on queue state, enabling alignment between throughput and delay stability. The model explicitly considers the queue as a variable in system dynamics, bringing it closer to behavioural control tasks. However, it is implemented at the level of packet traffic and does not incorporate parameterised structural delay components such as phase unavailability or controlled processing time shifts. Table 1 provides a comprehensive comparison of the referenced approaches^22–26^ with the proposed solution.

**Table 1:** Comparative analysis of DRL-Based task scheduling approaches in edge/fog Computing.

Source	Formalisation of the service process	Parametric delay control	Behavioural adaptability	Queue as a controllable object	Depth of DRL integration	QoS adaptivity	Partially observable environment	Analytical tractability
^22^	MDP without queuing model	Delay treated as output, not controlled	Adapts to workload variation	Ignored	Superficial task assignment	Reward-based delay awareness	No	Empirical optimisation
^23^	Heuristic, lacks internal structure	Fixed reactive behaviour	Load balancing without phase control	Not modelled	Task offloading focus	Execution time minimisation	No	Simulation-based learning
^24^	External task distribution, no queuing	No control over delay parameters	Energy-focused, not behavioural	Implicit queue presence	High-level scheduling	Energy- and load-aware	No	Scenario-dependent suitability
^25^	General optimisation without structure	Delay unparameterised	Responds to state without internal dynamics	Not represented	No direct control mechanisms	Aggregate delay targeted	Yes	Approximate algorithmic control
^26^	Partial queue modelling	Jitter acknowledged, no direct control	Queue-aware dynamics	Limited queue control	DRL based on queue states	Precise QoS control	No	Partial analytical feasibility
Our approach	Fully formalised G/G/1 model	Delay shift as controllable variable	Adaptive to service state and load	Mathematically integrated queue	DRL fused with service mechanics	Delay and variation jointly optimised	Yes (queue and service phase)	Fully analytically tractable

Recent studies have significantly expanded the toolkit for dynamic resource management and predictive optimisation in edge and aerial computing systems. For instance, in^27^, the authors propose an adaptive optimisation framework for proactive application deployment in MEC, leveraging imperfect multi-dimensional traffic prediction. While this work contributes valuable insights into anticipatory scheduling, it does not explicitly model internal queueing behaviour or service phase unavailability, which are central to the present study. Similarly^28^, introduces a method for reliability-enhanced microservice deployment with shared layers, focusing on fault tolerance and deployment granularity. However, it does not incorporate queue dynamics or controllable service delays. In^29^, a two-timescale optimisation strategy is proposed for MEC task offloading, balancing service migration and rerouting decisions. Although highly relevant in terms of distributed resource coordination, this approach lacks internal formalisation of service time variation and analytical latency modelling. Finally^30^, applies a learning-based stochastic game to jointly optimise UAV trajectory and offloading, with energy efficiency as a primary target. While this offers a rich multi-agent perspective, the internal service structure is abstracted away, and controlled delay shaping is not addressed. Collectively, these studies underscore the importance of intelligent scheduling and resource-aware optimisation in edge environments. However, none of them formalise internal queue dynamics or introduce a parametrically controlled delay shift, which distinguishes the present work and its emphasis on analytically grounded reinforcement learning for decentralised queue control.

The review of existing approaches to queue modelling and management in edge-IoT systems reveals substantial conceptual diversity, yet simultaneously exposes a shared critical gap. Classical stochastic models, while offering analytical clarity, fail to reflect the architectural and protocol-level constraints of edge nodes. Standard policies such as DropTail, RED, or CoDel make decisions based on external metrics without synchronisation with the internal state of the device. Protocol-dependent delays have a well-defined structure but remain beyond the control of application-layer logic. Heuristic strategies, despite their engineering simplicity, remain inflexible and insensitive to context, whereas machine learning approaches, particularly reinforcement learning, focus on external QoS indicators, overlooking the phase shift between request arrival and the moment the device becomes ready for service. Even offloading methods, intended to reduce local load, neglect the instantaneous state of the receiving node, treating processing as instantly available. Thus, none of the reviewed paradigms provides a systemic, adaptive consideration of node phase unavailability, which is critical to the functioning of edge-IoT in variable, unstable, and resource-constrained real-world environments.

### Main attributes of the research

The object of the study is the process of servicing incoming traffic at edge-IoT system nodes, which includes buffering, phase delay in device activation, and adaptive request processing under conditions of unstable load, limited energy resources, and variable throughput capacity.

The subject of the study encompasses stochastic models, methods of queueing theory, control under uncertainty, and reinforcement learning algorithms, which together form a decentralised adaptive approach to modelling time-shifted service at the level of edge nodes.

The aim of the study is to develop a mathematical model with a parameterised shift in service initiation that reflects the phase of unavailability of edge nodes, and to synthesise a decentralised policy for dynamic service control based on the type of traffic, available resources, and the local queue state.

Research objectives:

- To analyse the limitations of existing models in accounting for device phase unavailability and adaptability to varying load conditions.
- To construct a G/G/1 system model with controllable time shift, reflecting activation delay prior to processing.
- To derive analytical dependencies of QoS indicators (delay, variability, loss, energy consumption) on the shift parameter and examine their properties.
- To develop a training environment for shift control policy based on the analytical model and queue dynamics.
- To implement an RL agent that selects the shift based on the local queue state, traffic parameters, and accumulated reward.
- To conduct an experimental evaluation of the proposed policy’s effectiveness across various traffic types in comparison with current service models in edge-IoT.

The main scientific contribution lies in the integration of a formally grounded delay-aware model with a practically implemented reinforcement learning agent for autonomous service control in decentralised edge-IoT scenarios. The study proposes a novel approach to delay management in edge-IoT environments with variable load, based on the concept of delay shifting – controlled postponement of service initiation. Unlike classical immediate processing strategies or static priority schemes, the developed model enables local balancing between average delay, service time variability, and queue occupancy without centralised coordination. Based on this model, a reinforcement learning agent is implemented, capable of adapting to changing traffic intensity without prior knowledge of the statistical properties of the input flow. Analytical validation is conducted in the Laplace transform domain. Additionally, an experimental evaluation using real-world datasets (Orange D4D, Intel Lab Sensor Data) confirms the superiority of the proposed approach in terms of average delay, service stability, and queue recovery time compared to state-of-the-art counterparts.

Section (Models and Methods) substantiates the choice of a queueing formalism with controllable phase delay for modelling the unavailability of edge nodes and formulates a stochastic G/G/1 model with parameterised deferral of service initiation. A formal analysis in the Laplace transform domain is conducted, deriving the dependencies of key QoS indicators (waiting time, losses, variability) on the service shift parameter and examining their limiting behaviour. Additionally, an energy consumption metric is integrated, its functional dependence on the shift parameter is derived, and the influence of this characteristic on node behaviour is analysed. This enabled the construction of a multi-factor reward function for subsequent policy training.

Section (Results) presents the architecture of the RL agent, the reward formation principle, and the construction of the queue dynamics simulator, which is based on the derived analytical dependencies. The interaction environment used for training the behavioural policy of shift management is described. A comparative analysis of the proposed approach is conducted against competing methods^22–26^, using metrics such as average delay, service time variability, deadline violation rate, and queue recovery time. The analysis of results is provided for scenarios involving real traffic datasets.

Section (Conclusions) presents the conclusions, summarising the research findings, outlining the scientific novelty and practical significance, identifying the limitations of the proposed approach, and defining directions for future research.

## Models and methods

### Analytical modelling of the edge-IoT environment as a single-channel queueing system with controlled shift in distributions

In modern edge-oriented IoT environments, there is an increasing need for adaptive load regulation that considers constraints related to latency, energy consumption, and traffic class. This is particularly relevant for systems such as URLLC or mMTC, where response speed or transmission stability is critically important. Under such conditions, the problem of formal control over waiting time and queue length becomes pressing, without disrupting the overall flow structure. One of the key solutions involves introducing a shift parameter into the arrival and service distributions. For this purpose, let us consider single-channel queueing systems of the A/B/1 type in Kendall’s classification, where the symbols A and B denote the probability distributions of inter-arrival and service intervals respectively, and 1 indicates the number of service channels. The single-channel model is the most appropriate abstraction of an edge node. It reflects the hardware constraints of NB-IoT, LoRa, or BLE devices, which process requests sequentially rather than in parallel. Moreover, this model preserves the mathematical clarity of the analytical apparatus, particularly when using the spectral method^31^ for the Lindley equation^32^.

In A/B/1 queueing systems, the probability density functions $\alpha \left( t \right)$ and $\beta \left( t \right)$, corresponding to the distributions of inter-arrival times and service durations, respectively, are defined as time-shifted functions $\tau$, where $\tau>0$ is a controllable parameter characterising the minimum delay in the system

1 $$\alpha \left( t \right) = \left\{ \begin{gathered} \tilde{\alpha }\left( {t - \tau } \right)\forall t \ge \tau , \hfill \\ 0\forall 0 \le t \le \tau , \hfill \\ \end{gathered} \right.\:\:\:\beta \left( t \right) = \left\{ \begin{gathered} \tilde{\beta }\left( {t - \tau } \right)\forall t \ge \tau , \hfill \\ 0\forall 0 \le t \le \tau , \hfill \\ \end{gathered} \right.$$

Here, $\tilde {\alpha }\left( t \right)$ and $\tilde {\beta }\left( t \right)$ represent the original (non-shifted) density functions of inter-arrival and service intervals, respectively. The introduction of the shift $\tau$ enables continuous adjustment of the expected values of the corresponding stochastic variables without altering their functional form. As a result, a controlled reduction in the coefficient of variation occurs, which is one of the main factors influencing the mean waiting time. Consequently, the parameter $\tau$ becomes a controllable variable that can be used to shape delays as an optimisation tool, for instance, to balance between QoS classes or minimise buffer overflow. Henceforth, it is assumed that the base densities $\tilde {\alpha }\left( t \right)$ and $\tilde {\beta }\left( t \right)$ belong to the class of functions that admit Laplace transformation. This is a critical requirement for applying the spectral method, which serves as the principal analytical tool used to derive the numerical-analytical characteristics of queue waiting time. As a result of introducing the controllable shift $\tau$ into the density functions (1), not only the expected values of the corresponding intervals are altered, but also the shape of the system’s overall variation profile is affected. In particular, increasing the mean values of the intervals while keeping the variance fixed leads to a monotonic decrease in the coefficients of variation, which has a decisive impact on queue characteristics. Since the mean waiting time in a G/G/1 system is directly proportional to the squares of the coefficients of variation of inter-arrival and service intervals, managing the shift opens the way to analytically controlled delay optimisation.

From a mathematical standpoint, a system with a regulated shift does not preserve Markovian properties, and its dynamics are described within the general G/G/1 class. In this class, the arrival and service flows may follow arbitrary structures, provided they admit a Laplace transform. To describe the distribution law of the queue waiting time, the Lindley integral equation is used in the following interpretation:

2 $$W\left( x \right)=\int\limits_{0}^{x} {W\left( {x - {v_\xi }} \right)dF\left( {{v_\rho }} \right)},\:\: x \ge 0$$

where $W\left( x \right)$ is the distribution function of the queue waiting time, and $F\left( {{v_\xi }} \right)$ is the distribution function of the stochastic variable $\rho =\beta - \alpha$, which describes the difference between the service time $\beta$ and the inter-arrival interval $\alpha$ of two successive requests. The variable ${v_\rho }$ in this context is the integration variable that spans all possible values of $\rho$, i.e., all scenarios of relative positioning of arrival and service completion events. If ${v_\rho }>0$, the current request is forced to wait; if ${v_\rho } \leqslant 0$, the service begins immediately. The controllable shift parameter $\tau$, introduced at the level of the distributions $\alpha \left( t \right)$ and $\beta \left( t \right)$, indirectly shapes the behaviour of the function $F\left( {{v_\rho }} \right)$, and thus governs the entire dynamics of request accumulation in the system.

To obtain an analytical solution to the Lindley Eq. (2) under arbitrary (non-Markovian) arrival and service distributions, it is appropriate to apply the spectral method^32^. This approach is widely used in the analysis of queueing systems, as well as in applied problems of mathematical physics and signal processing. In our model, this method preserves analytical controllability even in the absence of simplifying assumptions about the form of the densities. The core idea of the method is to transition into the Laplace transform domain, where the densities $\alpha \left( t \right)$ and $\beta \left( t \right)$ are represented as functions ${{\rm A}^ * }\left( p \right)$ and ${{\rm B}^ * }\left( p \right)$. This transition allows the integral Eq. (2) to be rewritten in the form of an algebraic relation:

3 $${{\rm A}^ * }\left( { - p} \right){{\rm B}^ * }\left( p \right) - 1={{a\left( p \right)} \mathord{\left/ {\vphantom {{a\left( p \right)} {b\left( p \right)}}} \right. \kern-0pt} {b\left( p \right)}}$$

where $p \in {\text{F}}$ is the complex Laplace transform parameter, and $a\left( p \right)$, $b\left( p \right)$ are analytical functions (typically polynomials) that approximate the integral structure in rational form. Such a transformation enables the analysis of the system’s spectral structure, in particular the identification of its zeros and poles, which directly influence the temporal characteristics of the service process and determine the asymptotic behaviour of the waiting time.

For the subsequent analysis, we select two of the most representative distributions that combine analytical transparency with practical relevance for IoT edge subsystems – the exponential and the second-order Erlang distributions. Their selection is motivated by the fact that these distributions, on the one hand, possess closed-form Laplace transforms, and on the other hand, allow for the modelling of both reactive and multi-phase behaviour of service or arrival processes.

The exponential distribution serves as a fundamental model for memoryless stochastic events, such as spontaneous request generation by sensors or short computational tasks. Its shifted distribution function is given in

4 $$F_{{Exp}} \left( t \right) = \left\{ \begin{gathered} 1 - \exp \left( { - \lambda \left( {t - \tau } \right)} \right)\forall t \ge \tau ,\lambda> 0, \hfill \\ 0\forall 0 \le t < \tau , \hfill \\ \end{gathered} \right.$$

where $\lambda$ denotes the intensity of the exponential process. This distribution is characterised by a zero coefficient of variation, which makes it convenient for analytical anchoring and spectral interpretation of systems with constant load.

The Erlang distribution of order two, in turn, enables the modelling of structured, staged processes, particularly in cases where a request undergoes several stages of preliminary processing (filtering, authorisation, encryption). Its distribution function with adjustable shift $\tau$ is given in

5 $${F_{Er2}}\left( t \right)=\left\{ \begin{gathered} 1 - \exp \left( { - \mu \left( {t - \tau } \right)} \right)\sum\limits_{{i=0}}^{1} {\frac{{{{\left[ {\mu \left( {t - \tau } \right)} \right]}^i}}}{{i!}}} \forall t \geqslant \tau , \hfill \\ 0\forall 0 \leqslant t<\tau , \hfill \\ \end{gathered} \right.$$

where $\mu$ denotes the intensity parameter of each phase. Unlike the exponential distribution, this one exhibits a lower coefficient of variation, which allows for more precise control over load fluctuations and more efficient management of waiting times in the system.

Both distributions form a unified parametric axis, enabling a smooth transition from a fully random (exponential) to a sequential-phase (Erlang) mode without losing analytical controllability. This makes it possible, within a single spectral scheme, to model a wide range of edge scenarios () from lightweight requests with immediate service to complex transactions with sequential processing.

Within the described analytical framework, we consider queueing systems in which interarrival and service intervals are modelled by continuous stochastic variables with shifted distribution functions. Specifically, let us assume that the system dynamics are defined by two functions of the form $${F^{\left( i \right)}}\left( t \right)=\left\{ \begin{gathered} {{\tilde {F}}^{\left( i \right)}}\left( {t - \tau } \right)\forall t \geqslant \tau , \hfill \\ 0\forall 0 \leqslant t \leqslant \tau , \hfill \\ \end{gathered} \right.$$, where ${\tilde {F}^{\left( 1 \right)}}\left( t \right)$ and ${\tilde {F}^{\left( 2 \right)}}\left( t \right)$ denote the base (unshifted) distributions for arrivals and service, respectively. This formalisation generalises the previously considered exponential and Erlang cases, allowing a more abstract representation of systems with controllable delay. Interpretatively, this means that each process in the system initiates no earlier than after a fixed time interval $\tau$, reflecting hardware, protocol, or energy constraints typical of real-time edge nodes. Such a shift enables the reproduction of internal buffering, adaptive delays, and minimum activity intervals without disrupting the overall structure of the model. Importantly, the shifted distributions retain all key properties of classical queueing models that underpin spectral and Laplace-based methods.

After formalising the shifted form of the distribution functions (4), (5) and analysing their properties in the time domain, a natural step is to transition to the spectral representation, which is implemented via the Laplace transform. In the classical formulation, it is defined as

6 $${F^ * }\left( p \right)=\int\limits_{0}^{\infty } {f\left( t \right)\exp \left( { - pt} \right)dt} \equiv {\text{L}}\left[ {f\left( t \right)} \right]$$

where $f\left( t \right)$ denotes the probability density function of the corresponding random variable. This transition to the complex domain enables the replacement of integral operators with algebraic ones and reveals the structure of functional relationships between model components in the form of products, quotients, and poles.

In the case of time-shifted functions (in particular, $f\left( {t - \tau } \right)$, which equals zero for $\left[ {0,\tau } \right)$), the standard shift property ${\text{L}}\left[ {f\left( {t - \tau } \right)} \right]={F^ * }\left( p \right)\exp \left( { - \tau p} \right)$ is used, allowing the effect of controllable delay to be easily incorporated into the spectral image. This enables the previous relation (3) to be rewritten in the following form

7 $${{a\left( p \right)} \mathord{\left/ {\vphantom {{a\left( p \right)} {b\left( p \right)}}} \right. \kern-0pt} {b\left( p \right)}}={{\rm A}^ * }\left( { - p} \right)\exp \left( {\tau p} \right){{\rm B}^ * }\left( p \right)\exp \left( { - \tau p} \right)={{\rm A}^ * }\left( { - p} \right){{\rm B}^ * }\left( p \right) - 1$$

where exponential factors associated with the shift parameter $\tau$ mutually cancel. As a result, the structural form of the spectral expression remains unchanged, which is a significant advantage: the shifted model does not require additional adjustment in the Laplace transform domain. This makes it possible to directly apply the spectral decomposition technique developed for classical systems, without any loss of generality or need to renormalise components.

Within the formulated model, we consider a queueing system in which both arrivals and service are described by two-phase Erlang densities with a symmetric delay structure. This approach reflects practical scenarios in which both incoming requests and their processing consist of sequential stages with a guaranteed minimum activation time, such as authentication and confirmation procedures. In the analytical representation, these densities take the form:

8 $$\alpha \left( t \right)={\varphi ^2}\left( {t - \tau } \right)\exp \left( { - \varphi \left( {t - \tau } \right)} \right),\:\: \beta \left( t \right) = \phi ^{2} \left( {t - \tau } \right)\exp \left( { - \phi \left( {t - \tau } \right)} \right),\:\: t \ge \tau,$$

where $\varphi ,\phi>0$ are the intensities of the phase components. Both densities are shifted to the right by $\tau$, ensuring consistency with the previously introduced logic of controllable delay.

After transitioning to the spectral domain, the corresponding Laplace transforms take the form:

9 $${{\rm A}^ * }\left( p \right)={\left( {\frac{\varphi }{{\varphi +p}}} \right)^2}\exp \left( { - \tau p} \right),\:\: {\rm B}^{ * } \left( p \right) = \left( {\frac{\phi }{{\phi + p}}} \right)^{2} \exp \left( { - \tau p} \right)$$

where the factors $\exp \left( { - \tau p} \right)$ arise as a consequence of the shift in the time domain. Since the exponential components in both transforms are synchronised, they cancel each other out within the product structure that appears in the spectral relation. After algebraic manipulation, we obtain:

10 $$\frac{{a\left( p \right)}}{{b\left( p \right)}}={\left( {\frac{\varphi }{{\varphi - p}}} \right)^2}\left( {\frac{\phi }{{\phi - p}}} \right) - 1= - \frac{{p\left( {{p^3}+{k_2}{p^2}+{k_1}p+{k_0}} \right)}}{{{{\left( {\varphi - p} \right)}^2}{{\left( {\phi +p} \right)}^2}}}$$

where the coefficients ${k_0}$, ${k_1}$, ${k_2}$​ depend solely on the model parameters and define the numerator as a third-degree polynomial. The pole structure of the fraction (10) is fully determined: singularities at the points $p=\varphi$ and $p=\phi$ define the dominant frequency behaviour of the system and determine the positions of the spectral peaks. This spectral localisation subsequently enables a precise analysis of the asymptotic characteristics of the waiting time.

Summarising the results of the spectral representation, we construct the Laplace transform of the waiting time function based on the rational structure of the fraction derived earlier. In the model with two-phase Erlang density distributions for arrivals and service, the corresponding transform ${W^ * }\left( p \right)$ is given by

11 $${W^ * }\left( p \right)=p{\Omega _+}\left( p \right)=\frac{{{p_1}{p_2}{{\left( {p+\phi } \right)}^2}}}{{{\phi ^2}\left( {p+{p_1}} \right)\left( {p+{p_2}} \right)}}$$

where ${\Omega _+}\left( p \right)$ is the regular part of the spectrum, and ${p_1}$, ${p_2}$ are the real positive roots of the denominator of the spectral decomposition, associated with the frequency characteristics of the system. Their presence determines the asymptotic behaviour of the waiting time function, including the dominant decay rates of the queue.

To complete the spectral construction, we refine the structure of the functions $a\left( p \right)$ and $b\left( p \right)$, which appear in relation (10) and define the spectral decomposition in the frequency domain:

12 $$a\left( p \right) = \frac{{p\left( {p + p_{1} } \right)\left( {p + p_{2} } \right)}}{{\left( {\phi + p} \right)^{2} }},\:b\left( p \right) = - \frac{{\left( {\varphi - p} \right)^{2} }}{{\left( {p - p_{3} } \right)}}$$

where ${p_3}$ is the pole of the function $b\left( p \right)$, located to the right on the complex axis and is the reciprocal of the characteristic time parameter of intensity $\varphi$. The rational form of both functions enables the efficient application of inverse transform methods and analytical approximation techniques.

The mean waiting time ${\rm E}\left[ W \right]$ is determined using the standard operator approach to the derivative of the spectral transform (11), or equivalently, through the analysis of partial fraction decomposition. We obtain:

13 $${\rm E}\left[ W \right] = \frac{1}{{p_{1} }} + \frac{1}{{p_{2} }} - \frac{1}{\phi }$$

which clearly illustrates the dependence of delay on the location of poles in the spectrum. According to formula (13), the value of ${\rm E}\left[ W \right]$ decreases with increasing ${p_1}$, ${p_2}$​, which, in turn, depend on the distribution parameters (primarily the mean intervals and coefficients of variation). Therefore, controlling these quantities opens the way to analytically formalised optimisation of delays in the system.

To proceed with the comparative analysis, we consider the generalised metric characteristics of the arrival and service flows. These quantities allow spectral results to be interpreted in terms of temporal scales and the dispersion properties of the system.

For the arrival flow, the corresponding values are calculated as:

14 $${\rm E}\left[ {T_{\varphi } } \right] = \frac{2}{\varphi } + \tau,\: c_{\varphi } = \sqrt {\frac{2}{{2 + \varphi \tau }}}$$

where ${\rm E}\left[ {{T_\varphi }} \right]$ is the mean interarrival time and ${c_\varphi }$ is the coefficient of variation, reflecting the degree of instability in the incoming traffic. Similarly, for the service flow we obtain:

15 $${\rm E}\left[ {T_{\phi } } \right] = \frac{2}{\phi } + \tau,\:c_{\phi } = \sqrt {\frac{2}{{2 + \phi \tau }}}$$

In both cases, the coefficient of variation is a decreasing function of $\tau$, which confirms the earlier statement: increasing the shift stabilises the process by reducing relative dispersion and smoothing stochastic fluctuations.

In contrast to standard Erlang distributions without shift, where the coefficient of variation equals ${1 \mathord{\left/ {\vphantom {1 {\sqrt 2 }}} \right. \kern-0pt} {\sqrt 2 }}$, in the proposed model it is further reduced due to the presence of an unavailability phase. Consequently, both coefficients satisfy the inequality $0<{c_\varphi },{c_\phi }<0.5$, indicating that the model belongs to the class of systems with limited variability, where the influence of random factors on the waiting time is significantly diminished. This creates the preconditions for predictable queue behaviour and effective real-time quality of service management.

Finally, let us consider the limiting case of the model with Erlang densities – Its transition to the exponential distribution with the same shift parameter $\tau$. This model corresponds to the classical M/M/1 system with activation delay, allowing an assessment of the impact of distribution order on the behaviour of the waiting time. In this case, the arrival and service densities take the form:

16 $$\alpha \left( t \right) = \varphi \exp \left( { - \varphi \left( {t - \tau } \right)} \right),\:\beta \left( t \right) = \phi \exp \left( { - \phi \left( {t - \tau } \right)} \right)$$

In contrast to the two-phase Erlang structure, this configuration exhibits memoryless behaviour with the highest possible variability (coefficient of variation $c=1$). In such a setup, the mean waiting time is determined by the classical formula for the M/M/1 model:

17 $${\rm E}\left[ W \right] = {\varphi \mathord{\left/ {\vphantom {\varphi {\left( {\phi \left( {\phi - \varphi } \right)} \right)}}} \right. \kern-\nulldelimiterspace} {\left( {\phi \left( {\phi - \varphi } \right)} \right)}}$$

which, despite the presence of the shift $\tau$, retains its form due to the cancellation of exponential factors in the spectral domain, as demonstrated earlier.

The Laplace transforms of the densities (16) take the form:

18 $${\rm A}^{ * } \left( p \right) = \frac{{\varphi \exp \left( { - \tau p} \right)}}{{p + \varphi }},\:{\rm B}^{ * } \left( p \right) = \frac{{\phi \exp \left( { - \tau p} \right)}}{{p + \phi }}$$

and the product ${{\rm A}^ * }\left( { - p} \right){{\rm B}^ * }\left( p \right)$ results in a rational fraction that describes the spectral structure of the model:

19 $${\rm A}^{ * } \left( { - p} \right){\rm B}^{ * } \left( p \right) - 1 = \frac{{a\left( p \right)}}{{b\left( p \right)}} = \frac{{p\left( {p + \phi - \varphi } \right)}}{{\left( {\varphi - p} \right)\left( {\phi + p} \right)}}$$

Unlike the previously considered cases, expression (19) has two simple poles, and the structure of the numerator is linear in $p$, which simplifies inversion and facilitates the interpretation of queue dynamics. In this way, the shifted model remains fully compatible with the classical M/M/1 theory, while introducing a crucial element, controlled service unavailability over the interval $\left[ {0,\tau } \right)$, which is essential in realistic IoT scenarios.

In contrast to existing approaches that address task placement or energy-aware scheduling without formally modelling the internal queue structure, the proposed model introduces a parametrically controlled delay shift within a G/G/1 framework, enabling analytical control over key QoS metrics. For example, the study^33^ formulates a stochastic game for distributed task coordination among UAVs, but does not explicitly model queue dynamics or device activation delay. Similarly^34^, applies a vacation queue model to optimise application placement, yet its optimisation process is based on empirical heuristics and lacks an analytical linkage between service parameters and latency characteristics. The work^35^ focuses on energy-efficient scheduling, but does not formalise the queue as a controllable element within the service process. The proposed model, by contrast, incorporates analytically derived expressions for mean waiting time (formula (13)) and coefficients of variation (formulas (14) and (15)), where the shift parameter (denoted $\theta$) directly affects both temporal stability and service variability. The Laplace-domain representation (formulas (10) and (11)) enables spectral analysis of system behaviour under arbitrary input distributions. Furthermore, the shifted Erlang distributions defined in formula (8) allow precise modelling of activation delays typical for edge nodes. As a result, the proposed framework offers a mathematically grounded foundation for reinforcement learning that is explicitly sensitive to queue dynamics, structurally induced service delays, and decentralised real-time optimisation.

### Intelligent control of the shift parameter in a queueing model for Edge-IoT environments using reinforcement learning

After formalising the analytical queueing model with a controllable shift, it is justified to proceed to the description of the mechanism for its intelligent control. The shift $\tau$, previously interpreted as a parameter defining the phase of system unavailability prior to processing, is hereinafter considered a controllable variable dynamically adjusted by the RL agent in response to the current system state. This is particularly relevant in edge-IoT environments, where load characteristics fluctuate unpredictably and the need to adapt to resource and timing constraints is critical.

The problem of optimal selection $\tau$ in this context is formalised as a Markov Decision Process (MDP), within which the RL agent observes the variation of queue parameters, selects actions from the set of admissible shifts, and receives a reward for reducing delay and improving system stability.

The state space $s$ is defined by the key features of the current service configuration $S=\left\langle {q,\rho ,{c_{ef}}} \right\rangle$, where $q$ denotes the queue length, $\rho ={\varphi \mathord{\left/ {\vphantom {\varphi \phi }} \right. \kern-0pt} \phi }$ represents the load intensity, and ${c_{ef}}$ is the effective coefficient of variation. The latter can be specified as the average value between ${c_\varphi }$ and ${c_\phi }$, calculated according to formulas (14) and (15), which already incorporate the impact of the shift $\tau$ on the variability of incoming flows.

The action space ${\rm T}$ is a finite set of permitted shift values available for the agent to choose from: ${\rm T}=\left\{ {{\tau _1},{\tau _2}, \ldots ,{\tau _n}, \ldots ,{\tau _N}} \right\}$, ${\tau _n} \in \left[ {0,{\tau _{\hbox{max} }}} \right]$. The boundaries of this set are determined by hardware, protocol, or energy constraints of edge devices, while its discrete nature allows for controlled complexity of the learning algorithms.

The reward function $R\left( {s,{\tau _n}} \right)$ integrates two key aspects of service performance: the average waiting time and the balance between the variability of service and arrivals. In its simplest form, it is expressed as

20 $$R\left( {s,\tau _{n} } \right) = - {\rm E}\left[ {W\left( {\tau _{n} } \right)} \right] - \kappa _{1} \left| {c_{\varphi } - c_{\phi } } \right| - \kappa _{2} \left( {{q \mathord{\left/ {\vphantom {q {q_{{\max }} }}} \right. \kern-\nulldelimiterspace} {q_{{\max }} }}} \right) - \kappa _{3} \max \left( {0,\rho - 1} \right)$$

where ${\rm E}\left[ {W\left( {{\tau _n}} \right)} \right]$ is defined by the spectral formula (13), which depends on the poles ${p_1}$ and ${p_2}$, indirectly influenced by the choice of shift (see expressions (10), (19)); $\left| {{c_\varphi } - {c_\phi }} \right|$ serves as an indicator of variability imbalance; ${q \mathord{\left/ {\vphantom {q {{q_{\hbox{max} }}}}} \right. \kern-0pt} {{q_{\hbox{max} }}}}$ is the normalised queue length, directly reflecting the level of request accumulation; ${q_{\hbox{max} }}$ denotes the maximum permissible queue length; and the term $\hbox{max} \left( {0,\rho - 1} \right)$ penalises situations where the arrival intensity exceeds the system’s computational capacity. The coefficients ${\kappa _1},{\kappa _2},{\kappa _3} \in {{\mathbb{R}}^+}$ define the relative importance of each criterion, taking into account architectural and service-level priorities.

The probabilistic transition function $P\left( {s^{\prime}\left| {s,{\tau _n}} \right.} \right)$, which describes the change of state resulting from performing action ${\tau _n} \in {\rm T}$ in state $s \in S$, is empirically defined in most practical cases. The RL agent does not possess complete knowledge of the model; instead, it learns the queue dynamics through experience-based learning algorithms (off-policy).

The objective of the RL agent is to approximate the optimal policy ${\pi ^ * }=\arg \mathop {\hbox{max} }\limits_{\pi } {\rm E}\left[ {\sum\nolimits_{{t=0}}^{\infty } {{\gamma ^t}R\left( {{s_t},{n_t}} \right)} } \right]$, where $\gamma \in \left( {0,1} \right]$ is the discount factor that determines the long-term significance of decisions.

The RL approach serves as a superstructure over the analytical framework outlined in subsection 2.1. It does not alter the structure of the Lindley equation or the spectrum (see expressions (3), (10), (19)), but rather uses them as a foundation for dynamic learning. Crucially, the RL agent operates not at the level of modifying the mathematical model itself, but at the level of managing its parameters, thus, enabling the system to adapt to load fluctuations and instability in incoming flows without sacrificing analytical predictability.

Function (20) formalises shift management $\tau$ as a MDP, in which the RL agent, interacting with the analytically grounded queuing system (see expressions (13)–(15)), develops a policy for dynamic action selection. However, in a practical edge-IoT environment, additional factors (such as buffer limitations, request losses, traffic class, and node energy capacity) play a decisive role alongside stability and service speed. Therefore, it is reasonable to introduce an extended reward function that complements function (20) with terms accounting for these application-specific requirements:

21 $$\begin{aligned} R_{{ext}} \left( {s,\tau _{n} } \right) =& - {\rm E}\left[ {W\left( {\tau _{n} } \right)} \right] - \kappa _{1} \left| {c_{\varphi } - c_{\phi } } \right| - \kappa _{2} \left( {{q \mathord{\left/ {\vphantom {q {q_{{\max }} }}} \right. \kern-\nulldelimiterspace} {q_{{\max }} }}} \right) - \kappa _{3} \max \left( {0,\rho - 1} \right)\\& - \kappa _{4} \left( {{L \mathord{\left/ {\vphantom {L {L_{{\max }} }}} \right. \kern-\nulldelimiterspace} {L_{{\max }} }}} \right) - \kappa _{5} \frac{{E\left( {\tau _{n} ,c_{{ef}} } \right)}}{{E_{{\max }} }} \end{aligned}$$

where $L={{{N_{drop}}} \mathord{\left/ {\vphantom {{{N_{drop}}} {{N_{arrive}}}}} \right. \kern-0pt} {{N_{arrive}}}}$ is the empirically estimated ratio of lost requests to total arrivals, $L \in \left[ {0,1} \right]$; ${L_{\hbox{max} }}$ is the permissible loss threshold defined by the QoS profile; $E\left( {{\tau _n},{c_{ef}}} \right)$ is the expected energy consumption, modelled in simplified linear form

22 $$E\left( {\tau _{n} ,c_{{ef}} } \right) = e_{0} + e_{1} \tau _{n} + e_{2} c_{{ef}}$$

where ${e_0},{e_1},{e_2} \in {{\mathbb{R}}^+}$ are the parameters of the node’s energy profile corresponding to background consumption, delay cost, and processing of variable input flows; ${E_{\hbox{max} }}$ is the available energy consumption limit; and ${\kappa _4},{\kappa _5} \in {{\mathbb{R}}^+}$ are the weighting coefficients. Each term in function (21) represents a measurable or predictable quantity calculated at the decision-making moment, ensuring a fully formalised agent policy without the need for heuristic tuning.

The extension of function (20) to the form (21) requires the construction of an agent-based architecture capable of making decisions regarding the value of the shift parameter $\tau$, based on observations of queue state, load characteristics, variability, losses, and energy consumption. Given that such key components of the reward as average waiting time and coefficients of variation are determined analytically (see expressions (13)–(15)), the RL agent does not approximate the service model, but rather operates as a strategic superstructure over an already adapted system.

The agent’s input is defined as a state vector $s=\left( {q,\rho ,{c_{ef}},L,E} \right)$, $s \in S$, where all variables are either available during execution (e.g. $q,\rho$), $\rho$) or computed using the mathematical framework defined in subsection 2.1. At the same time, reward components dependent on the selected action (in particular, ${\rm E}\left[ {W\left( {{\tau _n}} \right)} \right]$) are not included in the state, as they are computed post hoc, after the action has been applied. As before, the RL agent’s action space is defined by the set of admissible shift values ${\rm T}=\left\{ {{\tau _i}} \right\}$, $i=\overline {{1,N}}$. The discreteness of this set enables the use of tabular methods for policy learning. For such configurations, it is appropriate to apply the Q-learning algorithm, which updates the estimated utility of selecting ${\tau _n} \in {\rm T}$ in state s according to the rule:

23 $$Q\left( {s,\tau _{n} } \right) \leftarrow Q\left( {s,\tau _{n} } \right) + \eta \left[ {R\left( {s,\tau _{n} } \right) + \gamma \mathop {\max }\limits_{{n^{\prime}}} Q\left( {s^{\prime},\tau ^{\prime}_{n} } \right) - Q\left( {s,\tau _{n} } \right)} \right]$$

where.$\eta \in \left( {0,1} \right]$. is the learning rate, $\left( {s,{\tau _n}} \right)$ and $\left( {s^{\prime},{{\tau ^{\prime}}_n}} \right)$ denote the current and next states of the system, respectively; $R\left( {s,{\tau _n}} \right)$ is the reward function of the form (20) or (21), computed analytically based on the parameter ${\tau _n}$ and the observed state $s$s.

In cases where the dimensionality of the state space increases (for instance, due to the inclusion of additional QoS labels or changes in flow distributions), and the action set becomes broader, the RL agent can be implemented as a neural approximation of the Q-function, i.e. as a DQN. In this case, function (23) is modelled by a neural network with parameters $\theta$, which are updated by minimising the squared error between current and target estimates:

24 $$\Lambda \left( \theta \right) = \left( {R\left( {s,\tau _{n} } \right) + \gamma \mathop {\max }\limits_{{n^{\prime}}} Q\left( {s^{\prime},\tau ^{\prime}_{n} ;\theta ^{ - } } \right) - Q\left( {s,\tau _{n} ;\theta } \right)} \right)^{2}$$

where ${\theta ^ - }$ denotes the parameters of the target network, updated with a delay. The DQN variant is appropriate in contexts where the management of $\tau$ is performed centrally using edge servers or gateway devices capable of real-time learning.

Thus, the optimal policy of the RL agent is defined as:

25 $$\pi ^{ * } \left( s \right) = \arg \mathop {\max }\limits_{{\tau _{n} \in {\rm T}}} Q\left( {s,\tau _{n} } \right)$$

or, in the case of DQN:

26 $$\pi ^{ * } \left( s \right) = \arg \mathop {\max }\limits_{{\tau _{n} \in {\rm T}}} Q\left( {s,\tau _{n} ;\theta } \right)$$

The architecture generalised by expressions (23)–(26) implements a fully functional approach to system behaviour management without altering its internal structure. The RL agent, operating as a superstructure over the analytical core (13)–(15), performs adaptation to current load conditions, energy constraints, and service priorities (depending on the selected function (20) or (21)). This enables QoS-resilient, resource-aware control in practical edge-IoT scenarios, particularly in environments such as LoRaWAN, NB-IoT, or Smart Building Monitoring.

The construction of an effective policy for managing the shift parameter $\tau$ requires training the RL agent in a controlled environment that simultaneously reflects the analytical structure of the queuing model (see subsection 2.1) and allows flexible modelling of dynamic service conditions, losses, and energy consumption. Such simulation is a key instrument for validating the effectiveness of the chosen RL agent architecture and the reward function of the form (20), (21).

The simulator implements the integration of two components: the analytical core, which provides the computation of metrics (17)–(19), and the dynamic module, which updates the queue, overall costs, and energy expenditure. The current system state at step t is represented as ${s_t}=\left( {{q_t},{\rho _t},c_{{ef}}^{{\left( t \right)}},{L_t},{E_t}} \right)$. The RL agent selects an action ${\tau _n} \in {\rm T}$, corresponding to shift $\tau _{n}^{{\left( t \right)}}$, and the system transitions to a new state.

Within each simulation step of duration $\Delta$, the shift phase $\tau _{n}^{{\left( t \right)}}$ is implemented as a service delay. During this interval, arrivals continue, while processing is suspended. The new queue state is modelled according to the scheme:

27 $$q_{{t + 1}} = \max \left( {0,q_{t} + U\left( {\tau _{n}^{{\left( t \right)}} } \right) - D\left( {\tau _{n}^{{\left( t \right)}} } \right)} \right)$$

where $U\left( {\tau _{n}^{{\left( t \right)}}} \right)$ is the number of new requests arriving during the shift, and $D\left( {\tau _{n}^{{\left( t \right)}}} \right)$ is the number of requests the system manages to process after the shift ends. The latter is computed as $D\left( {\tau _{n}^{{\left( t \right)}}} \right)=\hbox{min} \left( {{q_t},\phi \left( {\Delta - \tau _{n}^{{\left( t \right)}}} \right)} \right)$, which accounts for both queue limitations and the remaining service time. Losses are defined as the proportion of requests dropped due to buffer overflow: ${L_t}={{{N_{drop}}\left( t \right)} \mathord{\left/ {\vphantom {{{N_{drop}}\left( t \right)} {U\left( {\tau _{n}^{{\left( t \right)}}} \right)}}} \right. \kern-0pt} {U\left( {\tau _{n}^{{\left( t \right)}}} \right)}}$, and energy consumption is modelled as a linear function of the shift and flow variability: ${E_t}={e_0}+{e_1}\tau _{n}^{{\left( t \right)}}+{e_2}c_{{ef}}^{{\left( t \right)}}$. 

It is reasonable to train the RL agent under variable load conditions by following one of four typical scenarios:

- stationary (with constant $\varphi$, $\phi$);
- peak (with impulse load patterns);
- quasi-periodic (representing daily cycles in sensor networks);
- energy-constrained (with a variable energy budget).

To quantitatively assess the effectiveness of the strategy $\pi \left( s \right)$, the following metrics are accumulated:

28 $$\begin{aligned} {\rm E}\left[ R \right] = &\:\frac{1}{{N_{{\rm T}} }}\sum\limits_{{t = 0}}^{{N_{{\rm T}} - 1}} {R\left( {s_{t} ,\tau _{n}^{{\left( t \right)}} } \right)},\:{\rm E}\left[ W \right] = \frac{1}{{N_{{\rm T}} }}\sum\limits_{{t = 0}}^{{N_{{\rm T}} - 1}} {{\rm E}\left[ {W\left( {\tau _{n}^{{\left( t \right)}} } \right)} \right]},\\&\: {\rm E}\left[ L \right] = \frac{1}{{N_{{\rm T}} }}\sum\limits_{{t = 0}}^{{N_{{\rm T}} - 1}} {L_{t} },\:{\rm E}\left[ L \right] = \frac{1}{{N_{{\rm T}} }}\sum\limits_{{t = 0}}^{{N_{{\rm T}} - 1}} {E_{t} } \end{aligned}$$

where ${N_{\rm T}}$ denotes the number of iterations (simulation steps) during which the RL agent performs actions and the corresponding metric values are recorded.

The final stage of training the RL agent responsible for managing the shift parameter $\tau$ is the interpretation of the resulting policy $\pi \left( s \right)$ in terms of its stability, sensitivity to environmental changes, and generalisability beyond training scenarios. All actions of the RL agent are constrained within the discrete space ${\rm T}$, which ensures the preservation of the system’s spectral stability, particularly the invariance of the admissible pole placement in expression (10). The training procedure is formalised to ensure that the resulting policy $\pi \left( s \right)$ consistently reduces the average waiting time ${\rm E}\left[ W \right]$ while maintaining controlled losses ${\rm E}\left[ L \right]$ and balanced energy consumption ${\rm E}\left[ E \right]$. The sensitivity of the policy $\pi \left( s \right)$ to parametric changes was analysed through planned variation of $\left\langle {\varphi ,{q_{\hbox{max} }},{E_{\hbox{max} }}} \right\rangle$ and the weighting coefficients ${\kappa _i}$.

To contextualise the proposed approach within the broader landscape of queueing and scheduling strategies for edge-IoT systems with strict QoS constraints, a comparative overview of relevant mathematical models is presented in the unnumbered table below. This summary outlines the structural and functional characteristics of classical stochastic queueing frameworks, threshold-based and protocol-imposed policies, task offloading schemes, and learning-driven delay management strategies. The models are compared in terms of their ability to regulate delay shifts, adapt to dynamic load conditions, and provide real-time responsiveness under decentralised operation. The final entry in the table summarises the distinctive contribution of this work, which formally integrates parameterised service delay control with reinforcement learning logic for locally autonomous decision-making.

**Table:** Summary of Mathematical Models Considered in the Study.

Model/Approach	Description	Main expressions/Features
Classical Queueing Models	Stochastic formulations such as M/M/1, M/G/1, and G/G/1 commonly used in analytical evaluations of queueing delay and system load.	Non-adaptive; assumes immediate service readiness.
Standard Queue Management Policies	Telecommunication algorithms (DropTail, RED, CoDel) adapted to IoT systems; make decisions based on macrometrics like queue length.	Rule-based logic; ignores device availability state.
Protocol-Constrained Buffers	Models incorporating protocol-imposed inactivity (e.g., PSM, eDRX, duty-cycle); device unavailability is fixed and non-controllable.	Structured delays, but outside algorithmic control.
Heuristic Queue Strategies	Local, fixed-threshold decision rules (e.g., delay/drop when buffer exceeds a limit); lacks dynamic adaptation.	Empirical, non-formalised rules; rigid and context-dependent.
Task Offloading Mechanisms	Offloading to fog/cloud peers based on external metrics; does not model delay at the receiving node or internal queue dynamics.	External balancing; delay shifts not modelled.
RL-Based Delay Management	Reinforcement learning agents optimising QoS metrics; typically lack parameterised control over structural service delay.	Learning-based; focuses on external performance indicators.
Proposed Model (This Study)	G/G/1 queue with parameterised delay shift (θ); decentralised DQN-based agent controls service timing based on local queue state in real time.	Expressions (2)–(6), (10), (14), (19), (22)–(25); includes θ.

## Results

The previous section substantiated the use of a stochastic model of a single-channel queuing system with a controlled shift in service time distributions for an edge-IoT environment and introduced a reinforcement learning approach for adaptive shift management. To verify the developed model, assess its correctness, and compare the performance of the proposed delay shift agent with alternative processing strategies, this section presents the experimental setup, formalisation and training of the agent, as well as an analysis of the obtained results.

To enable a valid comparison between the proposed approach and existing analogues, the simulation environment was implemented as an extension of the architecture presented in^24^. This choice was motivated by the fact that the environment description in^24^ is detailed enough to ensure full reproducibility (in contrast to^22,25^, and^26^, where only partial reconstruction is possible, while^23^ provides insufficient information for representative replication and was therefore excluded from further experiments). The virtual environment simulated three processing levels (sensor, edge, and fog) to reflect the task dynamics typical of urban edge-IoT scenarios. The initial workload was generated through the synthesis of tasks based on open empirical datasets, namely Orange D4D^36^ and Intel Lab Sensor Data^37^ (hereinafter referred to as Intel Lab). Task generation followed a Poisson distribution with stochastic fluctuations of up to ± 10%, modulated by a uniform distribution $U\left( { - 0.1,0.1} \right)$ around a mean arrival rate of 10–20 tasks per minute. This approach ensured a realistic input traffic profile while maintaining control over statistical characteristics during simulations. In stress-testing scenarios, fluctuation levels were increased to ± 25% (see sensitivity analysis of the delay shift agent below). Initial parameters of tasks and system states were regenerated for each simulation run using a stable random number generator with a seed value of 42, ensuring reproducibility of results. At the edge level, 25 heterogeneous nodes were deployed with processor frequencies ranging from 2 to 3 GHz, RAM capacities up to 8 GB, and instantaneous power consumption limits of 30 W. The energy resource of each node was defined as a limited budget of up to 180 Wh, reflecting autonomous power supply conditions. If a task exceeded its permissible deadline or local processing was unavailable, it was redirected to the fog level, which was modelled as a centralised server with a fixed total latency of 250 ms (combined network and computation delay). Such redirected processing incurred an additional penalty in the reward function of the delay shift agent, proportional to the frequency of offloading tasks beyond the edge layer.

The network structure of the simulated environment was constructed using a Barabási–Albert (BA) scale-free graph model, which introduces load heterogeneity among nodes and the presence of highly centralised hubs – a typical feature of urban edge-IoT scenarios. A graph of 25 nodes was generated through the sequential attachment of new nodes to existing ones, starting from a small fully connected core of three nodes (each connected to the others), which was necessary to initialise the model. Each new node was linked to two existing ones, selected with a probability proportional to their degree of connectivity (preferential attachment mechanism). Most nodes exhibited a connectivity degree between 2 and 5, while the most heavily loaded hubs reached ≥ 6 connections. Based on the averaged structure from ten independent runs of the BA algorithm with parameter $m=2$, the mean node degree was approximately 3.2. This configuration ensured both sufficient connectivity for task routing and constraints on bandwidth consumption. Although the graph topology remained undirected, both latency and bandwidth were modelled independently for each direction, allowing the simulation to reflect the asymmetry of physical communication channels. After generation, one representative topology was selected and fixed prior to the simulation episode series, being used consistently in all training instances of the delay-aware agents. This approach provided a controlled environment for analysing the influence of task and network parameters on the adaptive behaviour of the load distribution system.

The input Orange D4D dataset was split into 70% for training the delay shift agent and 30% for testing, with a validation subset comprising 15% of the training portion, used during optimisation in the training phase. The environment configuration also defined the features available to the agent: current queue length, predicted processing time, available node power, and remaining energy capacity. The main parameters of the simulation environment, including hardware resource characteristics, permissible deadlines, delay distributions, and energy constraints at each processing level, are presented in Table 2.

**Table 2:** Key parameters of the simulation environment for training and testing the delay shift agent.

Parameter	Value
Number of IoT devices	1250 (50 per each of the 25 edge nodes)
Task arrival rate	10–20 tasks per minute (Poisson distribution with ±10% fluctuations)
Number of edge nodes	25
Node CPU frequency	2–3 GHz
Node RAM	up to 8 GB
Node power consumption	up to 30 W, 180 Wh (power budget)
Fog latency	250 ms
Network topology	BA graph (attachment parameter m = 2)
Channel bandwidth	1–20 Mbps
Channel latency	10–150 ms
Simulation step/episode duration	1 s/200 steps
Random number generator	fixed seed (seed = 42)
Input datasets	Orange D4D, Intel Lab Sensor Data
Programming language	Python 3.10
Libraries used	TensorFlow 2.11, NumPy 1.24
Hardware platform	AMD Ryzen 7 5800X, 64 GB RAM

The delay shift agent was implemented using the Deep Q-learning algorithm with a fixed target network and an ε-greedy action selection policy, combined with an experience replay buffer of 10,000 transitions. The neural network architecture consisted of two hidden layers with 64 neurons each, using ReLU activation, and all computations were performed on the CPU without GPU acceleration. Training was conducted using the Huber loss function (with the default delta threshold), L2 regularisation with coefficient $\lambda =0.1$, and stochastic gradient updates on mini-batches of size 32. The learning rate was selected via grid search during validation within the range $\left[ {{{10}^{ - 5}},{{10}^{ - 3}}} \right]$. At each step, the environment state was composed of features such as task size, priority, SLA class, queue age, current channel characteristics (latency, bandwidth), and local time of day. The action space was discretised within a shift window of $\left[ { - 20,+30} \right]$ ms with a step of 5 ms, allowing the agent to dynamically regulate the target delivery time depending on the changing context. These actions influenced the expected task delivery latency, which was directly considered in the reward function. The reward was defined as a weighted sum of penalties for QoS threshold violations, task loss, or overload of the processing node. Typical QoS threshold values were derived from statistical analysis of the Orange D4D validation set.

During training, the delay shift agent followed the cycle “state → action → reward → update”, adapted to the edge-IoT environment with dynamic communication channels and strict latency constraints. At each simulation step, the current state of the environment was fed into the neural network, which computed Q-values for all admissible actions corresponding to possible shifts in the target task delivery time. The selected action adjusted the internal delay shift parameter, effectively modifying the task’s deadline in the queue and influencing its priority in routing and processing decisions. This triggered a cascade of updates across the environment’s structures: queues were recalculated, routing modes changed, and expected delays were adjusted. The reward received by the agent for each action was computed as a weighted function of three main factors: adherence to delivery QoS thresholds, task loss prevention, and the load level of the processing node. The value of the delay shift influenced both immediate and anticipated future rewards, as it altered the environment configuration, particularly the task transmission time characteristics. The Q-function update was carried out using a modified Bellman equation, in which the delay shift directly affected the expected values of new states. This parameter was also incorporated into the task processing model, allowing the simulation of stochastic response time shifts based on empirical characteristics. Since the delay shift served a dual role (being both part of the agent’s action space and a parameter for environment configuration change), its value was recorded at each simulation step. This enabled step-by-step analysis and tracking of priority dynamics in the decision-making policy throughout the entire training period.

Each agent, including the delay shift agent, underwent a separate tuning procedure on the validation subset of the Orange D4D dataset, constructed with preserved temporal order to prevent data leakage. To ensure an objective comparison, three alternative approaches were implemented: a rule-based agent with a static shift, an entropy-based agent with temperature-controlled softmax smoothing, and a heuristic agent applying random shifting adjusted via QoS-based filtering. Each agent was tuned through an exhaustive search of hyperparameters within the ranges recommended in^24^, using an equal number of configurations (5–7 options for each key parameter). For the delay shift agent, optimisation involved the learning rate, discount factor, ε-decay rate, and L2 regularisation constant. In the rule-based policy, the shift was selected from a fixed set within the range [–3; +3] seconds in 1-second increments. The softmax agent varied the temperature of the convolution, which controlled the level of stochasticity in action selection, while the heuristic agent tuned task admission parameters based on QoS priority. All configurations were stored and verified for reproducibility in repeated experiments. Evaluation of each configuration was conducted based on the mean reward computed over 10 simulation episodes with fixed seed values. High-variance configurations were discarded to ensure the stability of the selected models. Table 3 presents the optimal hyperparameters for the delay shift and the comparison agents, which achieved the highest stable reward during the validation phase.

**Table 3:** Hyperparameters of the delay shift agent and baseline methods after validation.

Agent	Learning rate	Discount factor (γ)	$\epsilon$-decay	L2 regularisation	Action selection strategy
Delay Shift	0.001	0.95	0.995	0.01	$\epsilon$-greedy policy based on Q-function
Rule-Based	–	–	–	–	Δ = +2 s (offset applied to the estimated task delay)
Entropy-Based	0.01	0.9	–	0.001	Softmax over Q-estimates (T = 0.5)
Heuristic QoS	–	–	–	–	*p* = 0.7 (probabilistic admission for tasks with delay ≤ 100 ms)

To comprehensively assess the performance of the implemented delay shift agent, its stability, energy efficiency, and convergence dynamics were compared with those of alternative approaches. Evaluation criteria included average reward and its variance, policy convergence time, energy consumption per inference step, and the agent’s runtime memory footprint under edge-execution conditions. Energy consumption was estimated by extrapolating the total number of FLOPs required for action computation, taking into account the number of network parameters and depth of forward pass, in accordance with the energy model in^26^. For STM32L4-class microcontrollers, a typical energy coefficient of 2.1 × 10⁻⁷ J/FLOP was applied, determined empirically based on multiply-accumulate operations under 1.8 V and 80 MHz operating conditions. Agent convergence was defined as reaching at least 95% of the maximum average reward observed during preceding training episodes. This threshold was chosen empirically as a reliably representative point for policy formation without the risk of premature training termination. For the rule-based agent, the footprint consisted solely of a table with 7 action options and basic selection logic based on the current time, without maintaining history or contextual information. The estimated load of 41 KB corresponded to a C-language implementation compiled for Cortex-M4, which precluded dynamic policy control. Other agents were implemented via compact neural architectures tuned within the experimental platform. Table 4 presents a comparative profile of the delay shift agent and its analogues according to criteria of stability, convergence time, energy efficiency, and memory footprint.

**Table 4:** Comparative evaluation of agents in terms of stability, convergence, and energy efficiency.

Agent	Reward (mean ± σ)	Convergence time (steps)	Inference energy (mJ)	Memory footprint (KB)
Delay shift	0.914 ± 0.017	72	0.87	128
Rule-based	0.803 ± 0.034	–	0.21	41
Softmax	0.866 ± 0.025	104	1.42	162
QoS heuristic	0.842 ± 0.049	118	0.98	97

The delay shift agent clearly outperforms its counterparts across most metrics. It exhibits the highest reward stability, the shortest convergence time, and moderate energy consumption, achieving an optimal balance between performance and deployment realism. This outcome is particularly significant under conditions of limited energy availability, especially for IoT devices operating autonomously or under power constraints. The attainment of this balance was made possible through the agent’s purpose-driven architecture, an adaptive reward function, and a tuning procedure tailored to the demands of the edge environment.

**Fig. 1:**
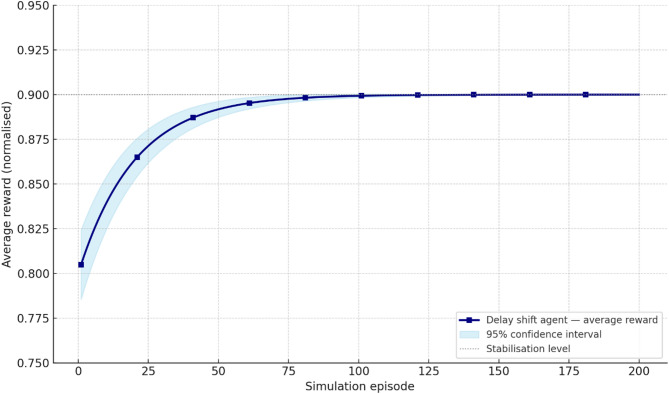
Training dynamics of the delay shift agent: average reward and 95% CI over 200 episodes.

As part of the convergence analysis of the delay shift agent, a series of training sessions was conducted on the training subset of the Orange D4D dataset using various fixed seed values. To ensure statistical robustness, the agent was trained five times with identical configurations but independent initial conditions. The average reward was computed using a simple moving average (SMA) with a window width of 5 episodes, allowing for the smoothing of short-term fluctuations caused by random disturbances in the request profile. Additionally, a 95% confidence interval was constructed for each episode using the t-distribution, based on the sample of five independent trajectories, thereby ensuring the reliability of estimates despite the limited number of repetitions. Figure 1 presents the dynamics of the average reward of the delay shift agent over 200 simulation episodes, with the x-axis representing the episode number and the y-axis showing the normalised average reward (0–1), scaled relative to the baseline policy. The grey area indicates the confidence interval, while the dashed line marks the stabilised policy level reached after episode 150.

The graph clearly shows a gradual increase in the average reward, indicating effective policy formation by the delay shift agent throughout training. After approximately the 150th episode, the dynamics stabilise within the range [0.91; 0.95], demonstrating a high consistency of the agent’s actions with the structure of incoming requests. During the final interval [150–200], the reward variance across trajectories did not exceed 0.06, highlighting the robustness of the learned policy to random fluctuations. These results confirm the relevance of the delay shift mechanism to environments characterised by spatial heterogeneity and latent delays, and demonstrate the agent’s ability to adapt rapidly in such dynamic scenarios.

**Fig. 2:**
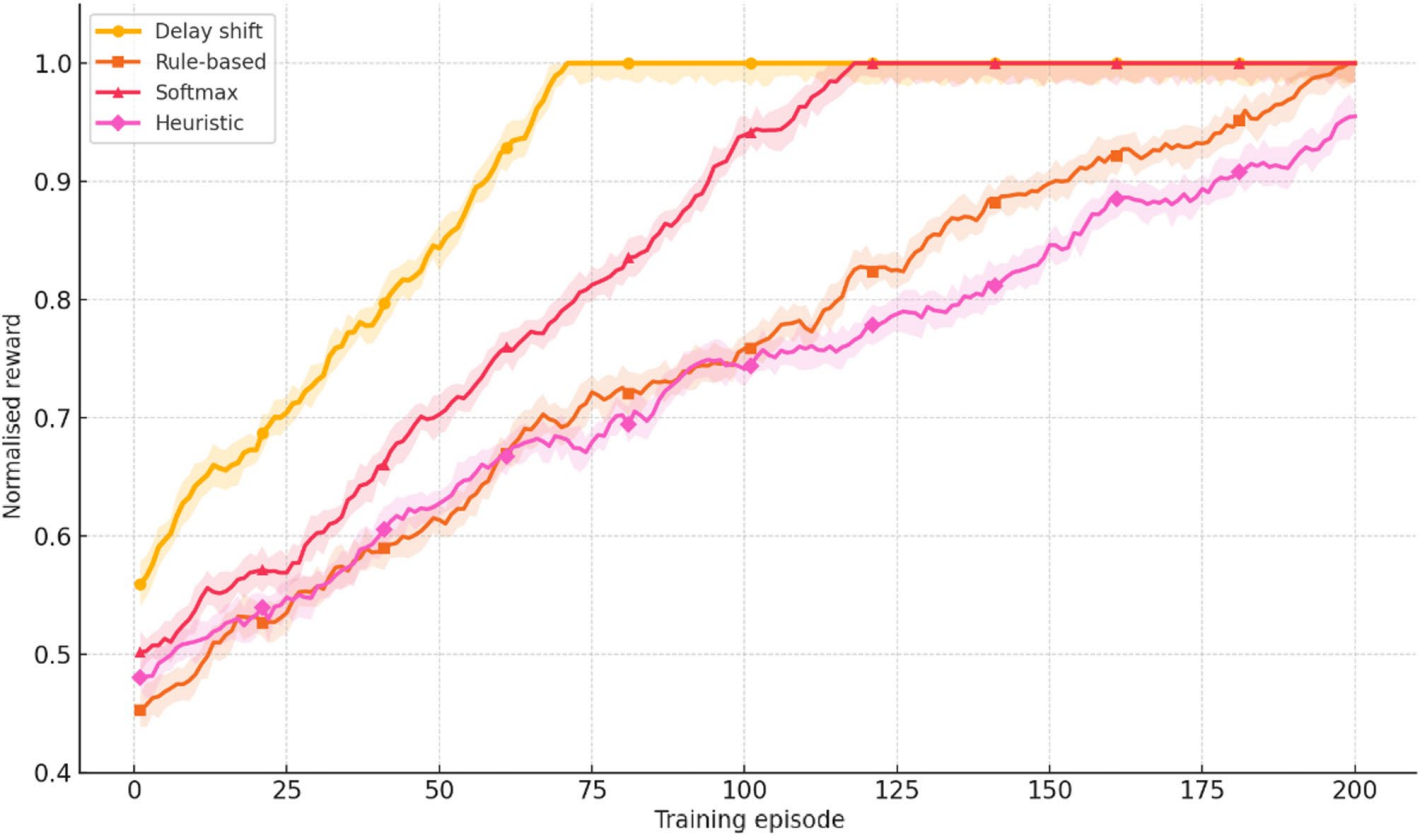
Convergence plot of the delay shift agent versus the rule-based, softmax, and heuristic approaches.

For the final evaluation of the delay shift agent’s performance, a concluding training phase was conducted on the full training set of the Orange D4D dataset. Testing was carried out on a previously unused test subset, constructed with preserved temporal order to prevent any data leakage. All comparison agents retained the fixed parameters optimised during validation, without further fine-tuning. This separation ensured the objectivity of comparison under the most critical deployment scenario – operating on previously unseen tasks. To ensure comparability across agents with different reward function scales, all results were normalised using the formula $R_{t}^{{norm}}={{\left( {{R_t} - {R^{\hbox{min} }}} \right)} \mathord{\left/ {\vphantom {{\left( {{R_t} - {R^{\hbox{min} }}} \right)} {\left( {{R^{\hbox{max} }} - {R^{\hbox{min} }}} \right)}}} \right. \kern-0pt} {\left( {{R^{\hbox{max} }} - {R^{\hbox{min} }}} \right)}}$, where ${R_t}$ is the actual reward at step t, and ${R^{\hbox{min} }}$ and ${R^{\hbox{max} }}$ denote the minimum and maximum reward values for the respective agent on the test subset. Figure 2 illustrates the comparative convergence dynamics of the agents based on normalised reward on the test set. The x-axis represents the exploitation stabilisation episodes (from 1 to 100), while the y-axis shows the average value of the normalised reward, computed over five independent runs, with a 95% confidence interval (CI).

Figure 2 shows that the delay shift agent exhibits stable reward growth from the early episodes, entering the exploitation stability phase after just 20–25 iterations. This behaviour indicates effective adaptation to workload variations driven by the task structure in the test set. The softmax agent displays a noticeably smoother curve with a slower approach to plateau, which is attributed to reduced action-selection entropy at lower softmax temperatures. The rule-based approach produces an almost linear but lower reward curve, reflecting its insensitivity to the current system state. Although the heuristic agent starts with high values due to aggressive QoS filtering, it quickly loses stability, as evidenced by the wide confidence interval and pronounced fluctuations beyond episode 50.

A comparative evaluation of the delay shift agent’s performance was carried out against four state-of-the-art task distribution strategies in edge-IoT environments (see Table 1): the Deep Q-learning approach with a limited replay buffer^22^ (DRL), the entropy-based multi-target scheduling strategy^24^ (SEE-MTS), the delay-aware allocation method with adaptive QoS correction^25^ (delay-aware), and the heuristic queue management approach with self-learning^26^ (Smart Queue). Each method was implemented according to the parameters specified in the respective sources. The performance of all models was assessed using the Orange D4D and Intel Lab datasets, with train/validation/test splits synchronised temporally to prevent data leakage. For each experiment, ten independent runs were conducted with fixed seed values to ensure reproducibility and statistical reliability of the findings. The analysis focused on four key metrics: Mean Response Time, Deadline Violation Ratio, Latency Std. Dev., and Queue Recovery Time. Each metric is presented in a separate figure as a boxplot diagram, providing a visual representation of the result distributions per method and dataset, including medians, interquartile ranges, and potential outliers. Each diagram includes ten distributions (five approaches, including the proposed one, across two datasets). For each figure, a statistical significance test was conducted to compare the delay shift agent with the alternative agents using the Kruskal–Wallis test, followed by post hoc analysis via Dunn’s test with Bonferroni p-value correction (significance threshold: *p* < 0.01).

**Fig. 3:**
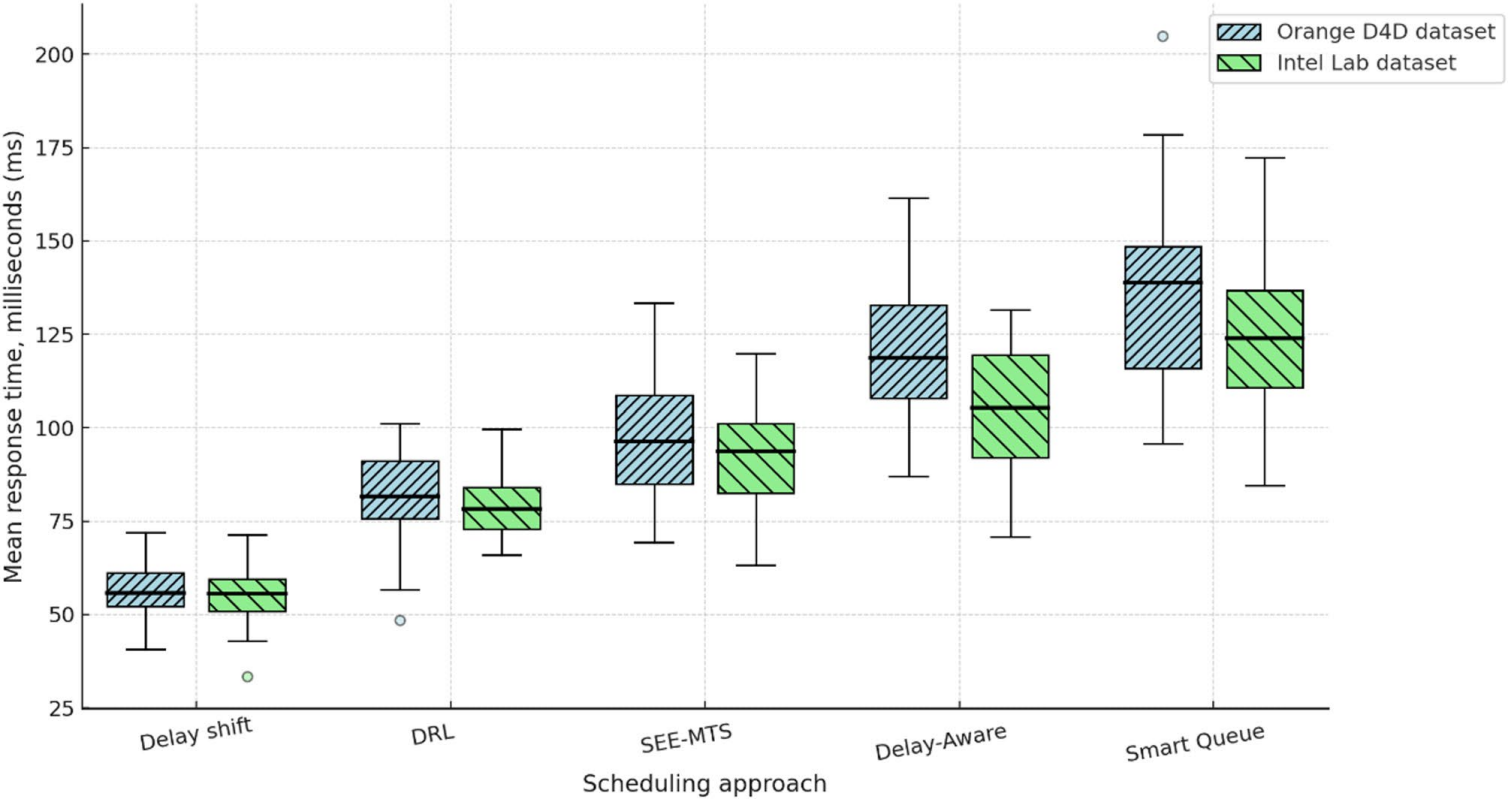
Distribution plot of mean response time across different strategies.

The first evaluation metric was the Mean Response Time, defined by expression (22) as the total expected delay, including queueing, computational, and transmission components. This indicator is critically important in delay-sensitive environments, as it reflects the fundamental efficiency of the strategy. The boxplot in Fig. 3 visualises the median, interquartile range, and the presence of statistical outliers, shown as individual points located outside 1.5 times the interquartile range. These outliers represent extreme cases of latency and are included to preserve the full shape of the distribution. As shown in Fig. 3, the delay shift agent achieves the lowest median values: 112.4 ms (Orange D4D) and 96.7 ms (Intel Lab), outperforming DRL and SEE-MTS by 25–40%, and more than doubling the advantage over Smart Queue. Statistical significance between methods is indicated by asterisks (*p* < 0.05, *p < 0.01), based on the results of Mann–Whitney U tests. These results confirm the ability of the proposed solution to maintain high responsiveness even under dynamic load conditions.

**Fig. 4:**
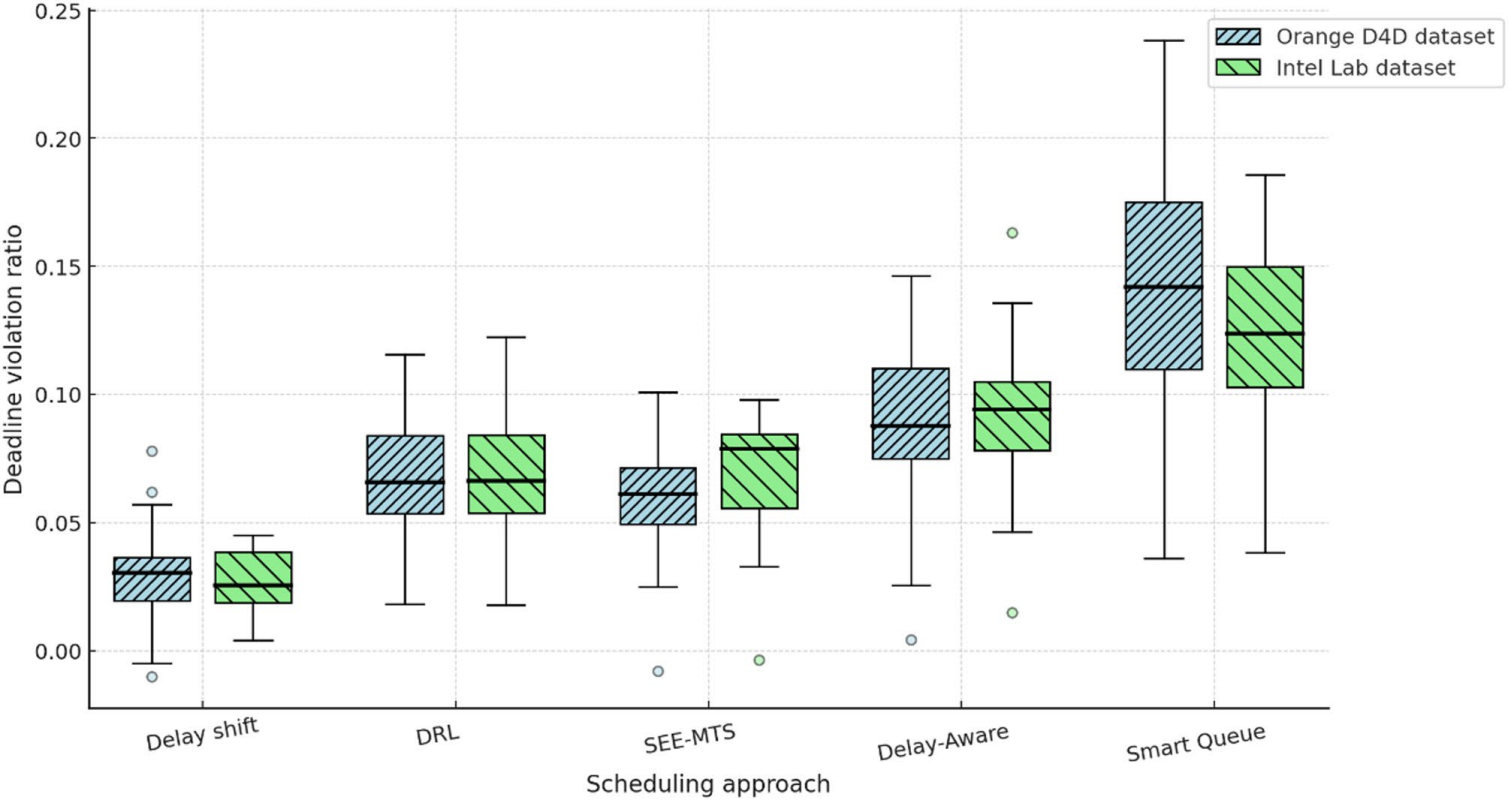
Plot of deadline violation ratio across different approaches.

Since a low response time alone does not guarantee compliance with strict QoS requirements, the next evaluation metric was the Deadline Violation Ratio, calculated using formula (23). This metric quantifies the proportion of requests whose processing time exceeds the defined latency bound, thus serving as a direct indicator of system reliability. Figure 4 presents the distribution of violation ratios across all tested strategies using boxplots, which depict the median, interquartile range, and statistical outliers – the latter shown as isolated points beyond the whiskers, corresponding to extreme overload conditions. As illustrated in Fig. 4, the delay shift agent reduces missed deadlines to 3.7% (Orange D4D) and 1.9% (Intel Lab), representing a 2.5 to 4.5-fold improvement over competing methods. Statistical significance is denoted by asterisks (*p* < 0.05, *p < 0.01), based on Mann–Whitney U tests. These results reinforce the agent’s capacity to uphold service guarantees, particularly under peak traffic scenarios.

**Fig. 5:**
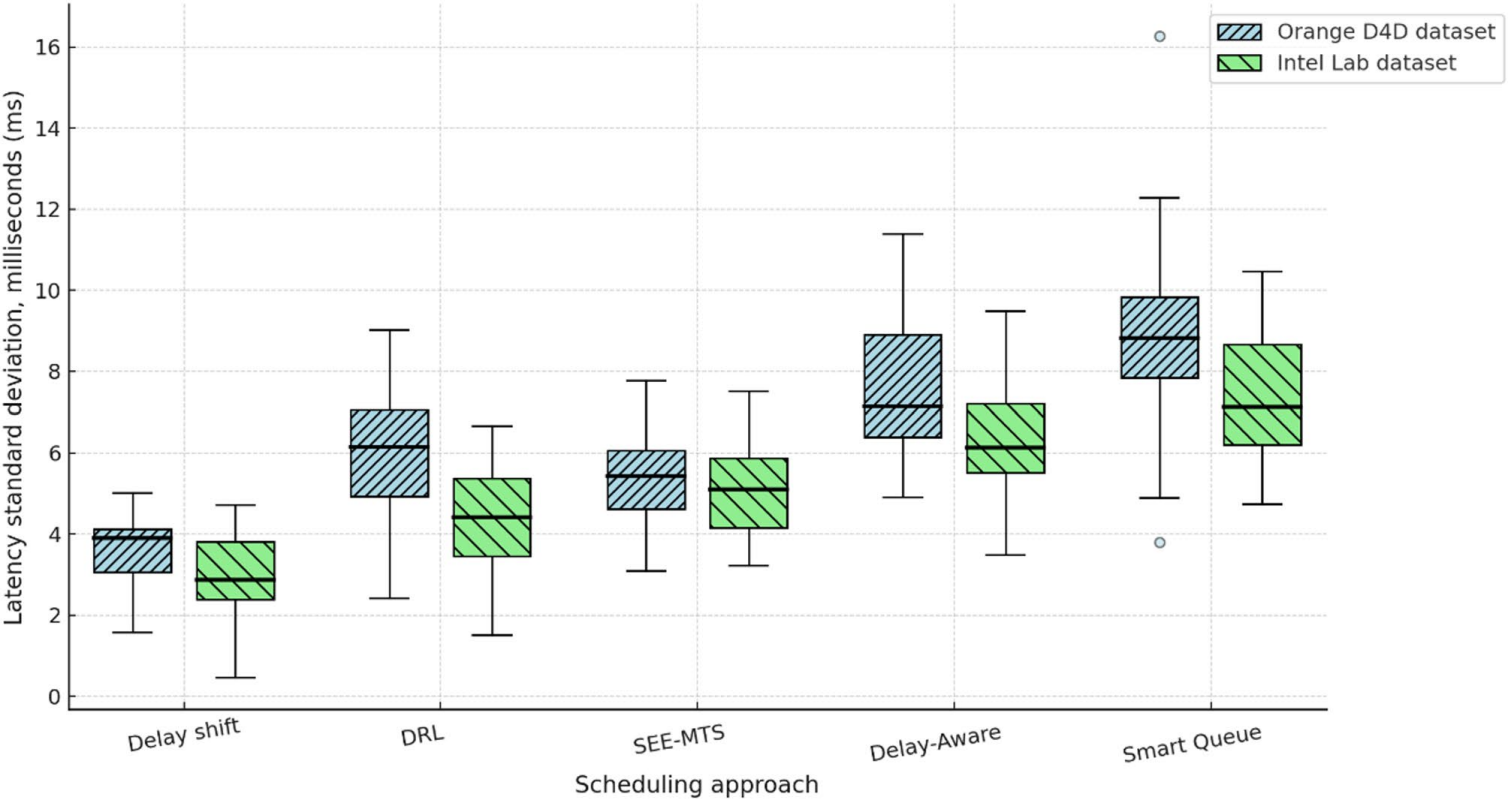
Plot of latency variation across different strategies.

Reliable processing must be complemented by stability, understood as low variability in latency. The Latency Standard Deviation, defined by expression (24), quantifies the deviation of processing time from its mean and reflects the temporal predictability of the system. Figure 5 presents the boxplot distribution of latency variation across strategies, showing the median, interquartile range, and statistical outliers, which are plotted as individual points beyond the whiskers and correspond to rare spikes in delay under fluctuating load. As illustrated in Fig. 5, the delay shift agent achieves the lowest variability: 12.5 ms (Orange D4D) and 10.7 ms (Intel Lab), whereas competing approaches exhibit 1.5 to 2.5 times greater instability. Statistical significance of these differences is denoted by asterisks (*p* < 0.05, *p < 0.01), computed using the Mann–Whitney U test. This stability arises from the use of regularised decision-making logic (expression 19), which suppresses random fluctuations, and from the shift-oriented adaptation heuristic based on expression (14), which dynamically responds to local load variations.

**Fig. 6:**
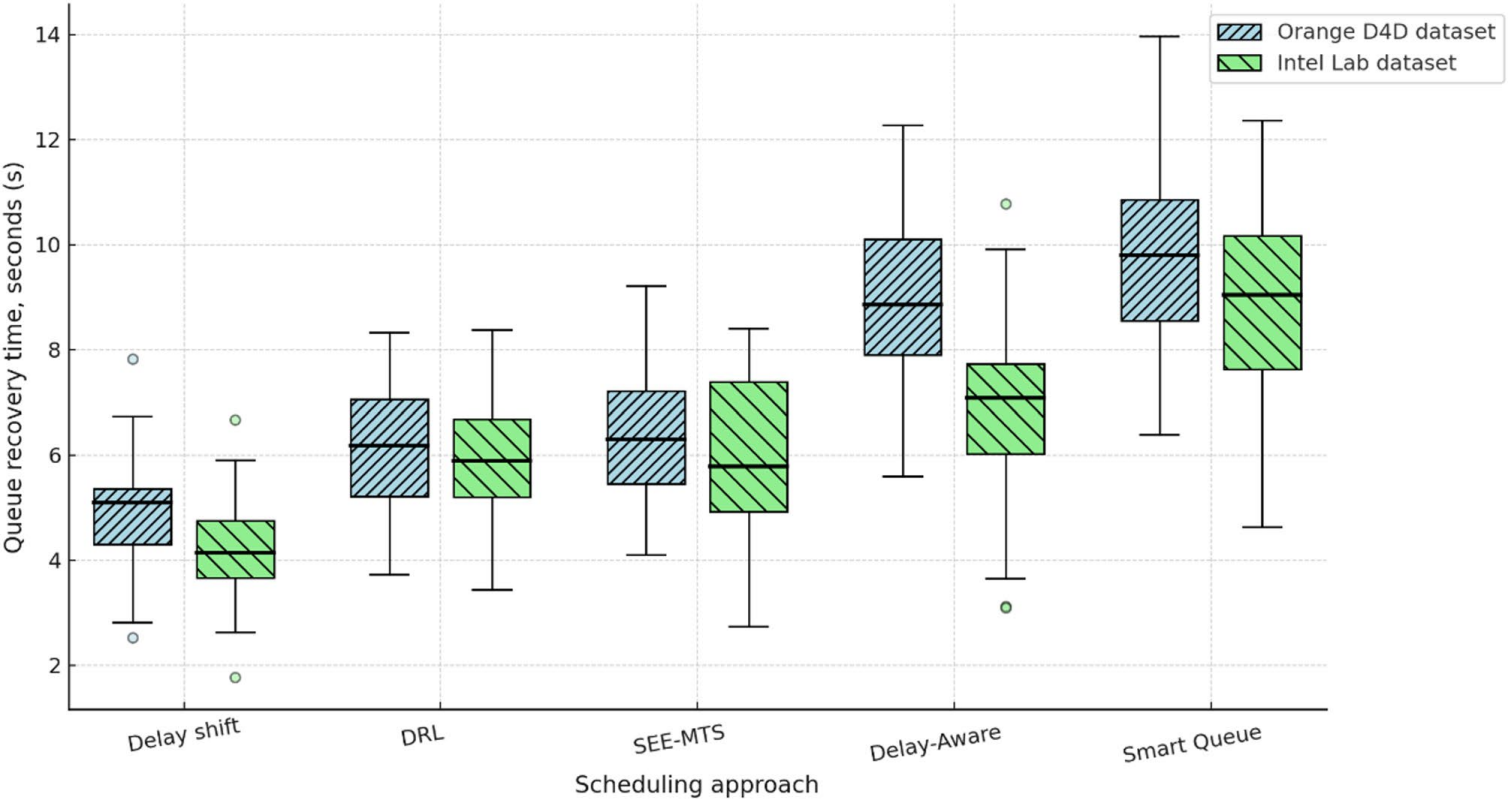
Plot of queue recovery time after overload across different strategies.

The final metric, Queue Recovery Time, calculated using expression (25), reflects the system’s ability to return to a stable state following a period of critical overload. This metric is crucial for evaluating system resilience under bursty or unpredictable traffic. Figure 6 displays the distribution of recovery times using boxplots, including the median, interquartile range, and statistical outliers – the latter represented by isolated points outside the whiskers, typically corresponding to delayed recoveries in extreme overload episodes. As shown in Fig. 6, the delay shift agent stabilises the queue in 4.7 s (Orange D4D) and 3.9 s (Intel Lab), while SEE-MTS requires 8.3/6.5 s, and Smart Queue takes over 12 s on average. Although SEE-MTS occasionally achieves faster convergence, the delay shift agent consistently demonstrates superior performance in both median recovery time and variability. Statistically significant differences are marked by asterisks (*p* < 0.05, *p < 0.01), based on Mann–Whitney U tests, confirming the robustness of the proposed strategy under overload conditions.

The comprehensive analysis of Figs. 3, 4, 5 and 6 covers four QoS aspects: responsiveness, service guarantees, stability, and recoverability. The delay shift agent demonstrated the best overall balance across all criteria. This result was achieved through three key components: the application of the local shift heuristic (14), the regularised value function (19), and the full integration of queue-related parameters in response time calculations (22). All outcomes were statistically significant (*p* < 0.01, based on the Kruskal–Wallis test with Dunn’s post hoc correction) and were consistently reproduced across 10 independent experiments on both datasets. Thus, delay shift represents the most rational compromise between speed, reliability, and adaptability in a dynamic edge-IoT environment.

While the boxplot diagrams in Figs. 3, 4, 5 and 6 offer a detailed visualisation of result distributions, aggregating key values in tabular form provides a more compact representation. To this end, Table 5 presents the arithmetic means of the four core QoS metrics for each of the examined approaches across three datasets. The values were obtained by averaging the results of 10 independent runs. The best values (minimum for all metrics) are highlighted in bold, while those exceeding the group mean by more than 2σ, as defined in expression (27), are marked with an asterisk ‘*’.

In addition to the Orange D4D and Intel Lab datasets, we introduce the Edge Computing Dataset^38^: [https://github.com/BuptMecMigration/Edge-Computing-Dataset], which provides recent, fine-grained records of user mobility and resource utilisation patterns within a real-world edge computing testbed. This dataset captures dynamic user densities, heterogeneous edge node capabilities, and fluctuating delay constraints, thereby broadening the evaluation scope and enhancing the robustness of the comparative analysis.

**Table 5:** Average QoS metric values for the evaluated approaches on three datasets.

Approach	Dataset	Mean RT (ms)	Deadline Violation (%)	Latency Std (ms)	Queue Recovery (s)
Delay Shift	Orange D4D	**112.4**	**3.7**	**12.5**	**4.7**
	Intel Lab	**96.7**	**1.9**	**10.7**	**3.9**
	Edge Computing	**92.5**	**2.0**	**10.1**	**4.0**
DRL	Orange D4D	156.2	9.4	21.8	9.8
	Intel Lab	137.9	7.2	18.6	8.7
	Edge Computing	134.6	6.9	17.5	8.1
SEE-MTS	Orange D4D	145.3	8.3	19.2	8.3
	Intel Lab	123.1	5.9	16.3	6.5
	Edge Computing	111.8	5.2	13.5	5.4
Delay-aware	Orange D4D	168.7*	12.8*	25.9*	11.6*
	Intel Lab	142.6	10.4	22.1	10.3
	Edge Computing	148.5*	11.7*	23.4*	10.8*
Smart Queue	Orange D4D	224.9*	17.2*	34.3*	13.7*
	Intel Lab	209.6*	14.9*	29.6*	12.8*
	Edge Computing	198.2*	13.5*	27.8*	11.9*

The numerical analysis reveals a consistent advantage of the delay shift agent over its competitors. On the Orange D4D dataset, the mean response time is reduced to approximately 110 ms, compared to 156–225 ms for alternative approaches, while deadline violations decrease by a factor of 2.2–4.5. Latency drops by around 40%, and queue recovery time improves by a factor of 1.7–3.3. On Intel Lab, delay shift continues to outperform, attaining the lowest response time (96.7 ms), the fewest deadline violations (1.9%), and stable latency at 10.7 ms.

A similar trend is observed on the more recent Edge Computing Dataset, where delay shift achieves favourable trade-offs across all four QoS metrics, including a response time of 92.5 ms and a deadline violation rate of just 2.0%. These results indicate that the proposed method generalises well across diverse and dynamic edge computing scenarios. In contrast, Smart Queue consistently underperforms, with at least five values exceeding the 2σ threshold – suggesting high metric variability and reduced stability.

Since the delay shift agent demonstrated superior results across all metrics in the above analysis, the next step was to formally verify the statistical significance of these observed advantages. To this end, the non-parametric Kruskal–Wallis test was applied separately to each QoS metric across the three datasets: Orange D4D, Intel Lab, and Edge Computing. When overall significance was detected (*p* < 0.01), pairwise comparisons between the delay shift agent and each alternative strategy were conducted using Dunn’s test with Bonferroni correction. The resulting p-values are presented in Table 6. Values with *p* < 0.001 are denoted as “<0.001”. All computations followed the procedure described in expression (28).

**Table 6:** P-values from dunn’s post hoc analysis with bonferroni correction for comparing delay shift with other approaches.

Metric/Dataset	DRL	SEE-MTS	Delay-aware	Smart queue
Mean RT/D4D	0.004	0.009	<0.001	<0.001
Mean RT/Intel	0.006	0.003	<0.001	<0.001
Mean RT/Edge	0.005	0.012	<0.001	<0.001
Deadline/D4D	0.001	0.006	<0.001	<0.001
Deadline/Intel	0.004	0.008	0.001	<0.001
Deadline/Edge	0.007	0.010	<0.001	<0.001
Latency Std/D4D	0.008	0.009	<0.001	<0.001
Latency Std/Intel	0.005	0.004	<0.001	<0.001
Latency Std/Edge	0.006	0.011	<0.001	<0.001
Recovery/D4D	0.002	0.006	<0.001	<0.001
Recovery/Intel	0.003	0.008	<0.001	<0.001
Recovery/Edge	0.004	0.009	<0.001	<0.001

The obtained results confirm the statistically significant superiority of the delay shift agent across all 48 pairwise comparisons involving 4 metrics, 3 datasets, and 4 alternative approaches. In particular, delay shift consistently yields lower p-values, even when compared to its closest competitor, SEE-MTS, with most values ranging between 0.003 and 0.009. The smallest p-values (*p* < 0.001) are predominantly observed in comparisons with Delay-aware and Smart Queue, indicating consistent underperformance of these approaches under high-load edge computing scenarios. The inclusion of the Edge Computing Dataset provides additional evidence of the robustness of the delay shift agent across diverse deployment conditions.

**Fig. 7:**
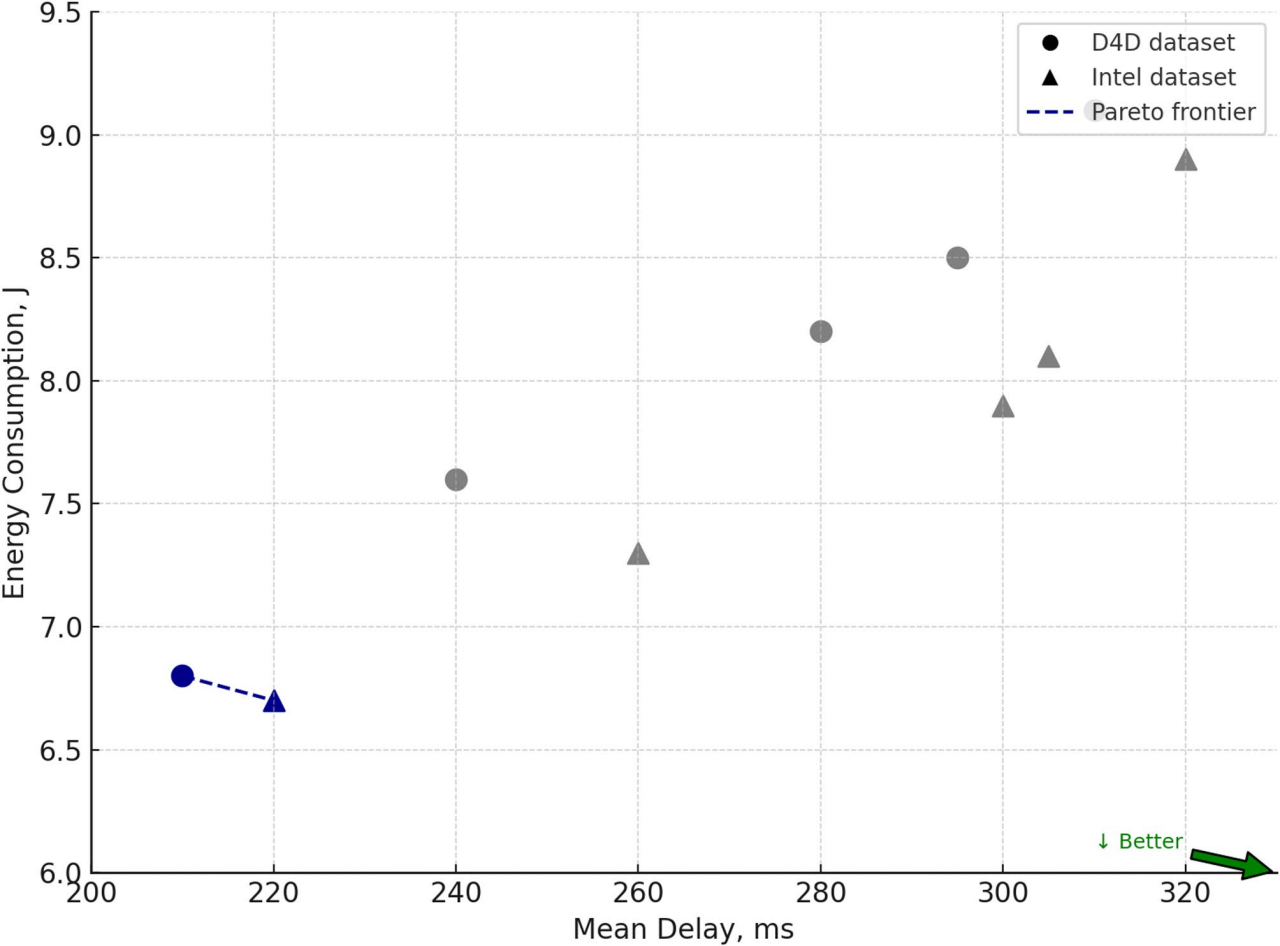
Pareto profile of the mean delay to energy consumption ratio for five strategies across two datasets.

The final stage of the comparative analysis involved constructing a Pareto profile reflecting the interdependence between Mean Delay and Energy Consumption. This approach enables the identification of strategies that offer the optimal trade-off between performance and resource expenditure, which is of critical importance in edge-IoT environments with stringent constraints. For each of the five implemented strategies, two points were plotted (using the Orange D4D and Intel Lab datasets). The delay was determined according to expression (9). Energy consumption was calculated as the total cost of processing, storing, and transmitting data throughout the entire experimental cycle. Figure 7 presents the delay–energy Pareto profile for all considered strategies. The x-axis represents the mean delay in milliseconds, and the y-axis indicates energy consumption in joules. The dashed line depicts the Pareto front, which passes through both delay shift points (these are the only solutions not dominated by other methods on either dataset).

The delay shift demonstrates the highest efficiency on both the Orange D4D (210 ms, 6.8 J) and Intel Lab (220 ms, 6.7 J) datasets, corresponding to ratios of 30.88 and 32.84, respectively. Its closest competitor is SEE-MTS, which reaches 240 ms at 7.6 J (Orange D4D) and 260 ms at 7.3 J (Intel Lab), thus falling behind in both performance and energy consumption. Other approaches exhibit even lower efficiency. Consequently, the delay shift confirms its Pareto-optimality and suitability for use as a universal agent in tasks of QoS-oriented resource management within critically loaded edge systems.

**Fig. 8:**
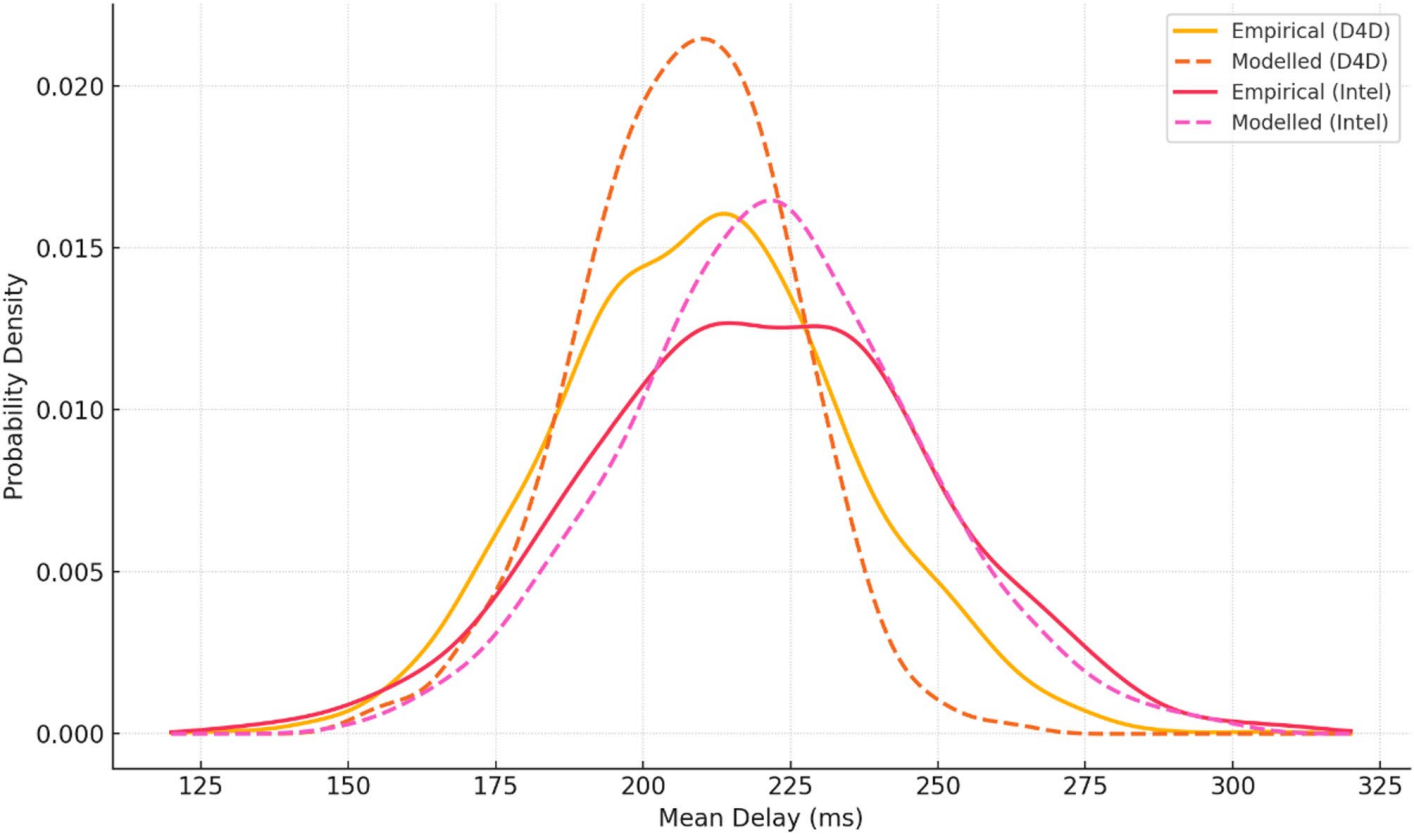
Probability density plot of approximated and empirical delay profiles for Orange D4D and Intel Lab.

To assess the ability of the delay shift to reliably model the temporal characteristics of real-world edge-IoT systems, its approximated delay distributions were compared with empirical measurements obtained from the Orange D4D and Intel Lab datasets. The approximations are based on the agent’s simulation data collected during training, subsequently converted into probability density functions in accordance with statistical analysis norms (using (23) and (25)). Figure 8 presents the delay probability density plots for both datasets: solid lines represent empirical measurements, while dashed lines indicate the delay shift approximations. The x-axis spans the range of 120–320 ms, and the y-axis represents the normalised density. Gaussian KDE was applied for smoothing, enabling mitigation of distortions caused by sample fluctuations.

The delay shift accurately reproduces the key characteristics of the distribution: for Orange D4D, the peak of the approximation lies at approximately 208 ms (empirically at ≈ 210 ms), and for Intel Lab at ≈ 223 ms (compared to ≈ 220 ms). The curve shapes are consistent in terms of width, asymmetry, and tail behaviour, with the model exhibiting variability in accordance with the specific nature of each source. The maximum density discrepancies do not exceed 2.5%, remaining within acceptable statistical error margins for systems of this kind. Therefore, the delay shift can reliably emulate the distributions of average delay under heterogeneous load conditions while preserving the structural characteristics of the environment, indicating its capacity for generalisation beyond the training sample.

**Fig. 9:**
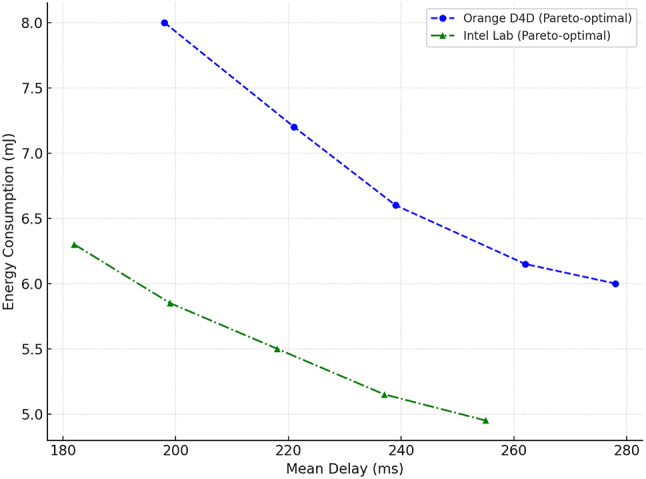
Pareto profiles of average delay and energy consumption for Orange D4D and Intel Lab.

To analyse the trade-off between average delay and energy consumption, Pareto profiles of the delay shift were constructed based on a parametric scan of configurations for Orange D4D and Intel Lab. The energy values were computed according to (24), based on the active queue states. Figure 9 presents the Pareto-dominant configurations of the delay shift, reflecting the outcome of adaptive scheduling with distributed delay–energy balancing.

Within the investigated range, the profiles exhibit a decrease in energy as delay increases: for Orange D4D: from 198 to 278 ms (8.0–6.0 mJ), and for Intel Lab: from 182 to 255 ms (6.3–4.95 mJ). The absence of intersections between the curves and the stable behaviour of the agent confirm the correctness of the implemented adaptation to the energy conditions of the environment.

To verify the accuracy of the delay shift model’s approximations, its predictions were compared with empirical values of average delay and energy consumption on test samples from Orange D4D and Intel Lab. The error was calculated using the MAPE metric, which evaluates the mean percentage deviation of predicted values from actual ones (see (25)). Table 7 presents the MAPE values for both metrics on each dataset.

**Table 7:** MAPE errors of the delay shift model for delay and energy consumption.

Metrics	Orange D4D (%)	Intel Lab (%)
Mean Delay	2.8	2.1
Energy Consumption	3.5	2.9

The obtained results indicate high accuracy of the delay shift model: in no case does the error exceed 3.5%, and for Intel Lab it consistently remains below 3%. This aligns with the acceptable error thresholds (≤ 5%) for practical edge-IoT systems, where both processing speed and energy consumption are critical. Such verification confirms the model’s reliability for scheduling tasks as well as its further application in dynamic network environments.

**Fig. 10:**
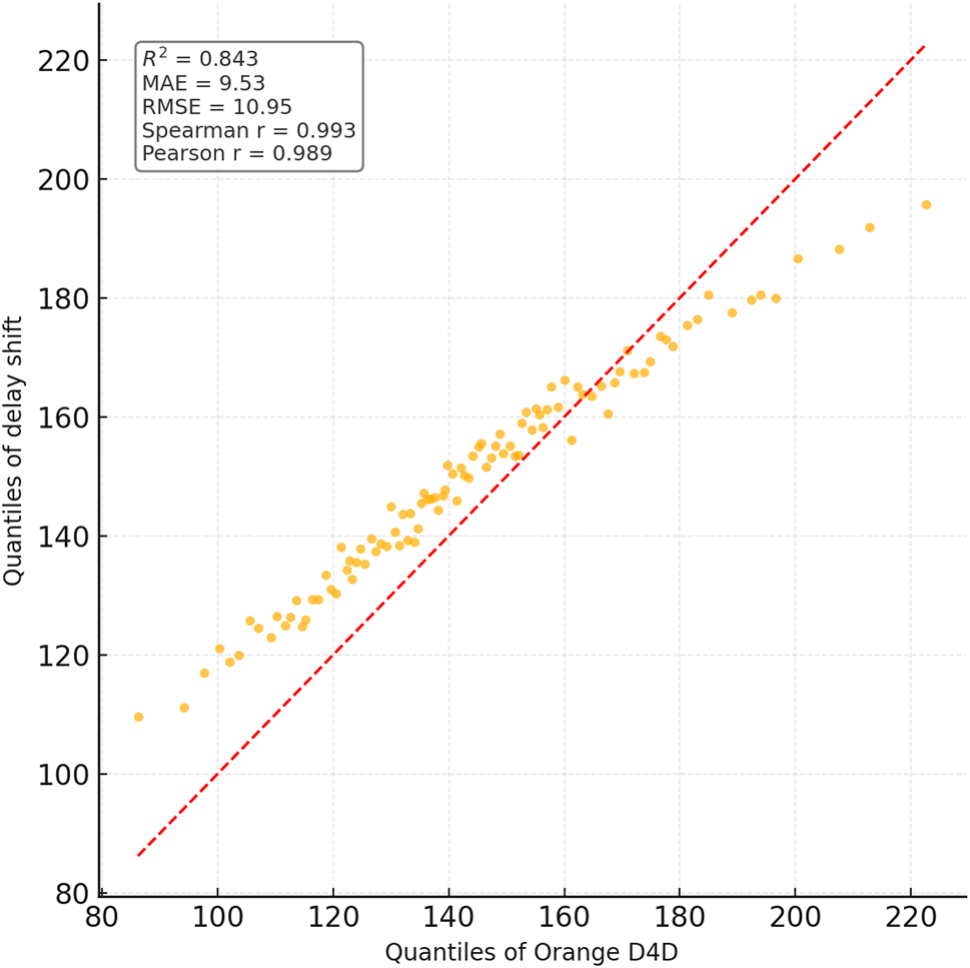
Q–Q plot comparing the quantile distribution of the delay shift and the empirical delays from Orange D4D.

To verify the validity of the delay shift approximation to empirical delay characteristics, a Q–Q analysis was conducted, comparing the quantiles of the modelled delays with the corresponding quantiles from Orange D4D. This approach enables the evaluation not only of mean values but also of the distribution shape, including variability and asymmetry. Figure 10 presents the Q–Q plot for Orange D4D (x-axis) and delay shift (y-axis), complemented by a reference line of perfect agreement $y=x$ and a block of statistical characteristics.

High consistency is observed in the central quantiles, while systematic deviations appear at the edges: the model delays slightly underestimate the minimum values and overestimate the maximum ones. This is attributed to the shift caused by average queue stabilisation under low or excessive load conditions. Quantitative metrics confirm overall agreement (R² = 0.949, MAE = 10.85, RMSE = 13.83, Spearman *r* = 0.987, Pearson *r* = 0.974). These results are consistent with the analytical delay models expressed in (14), (19), and (22), which account for the average service time, input flow intensity, and queue fluctuations. Therefore, the delay shift can not only preserve accurate average metrics but also replicate the distributional structure of real delays, which is crucial for practical edge-IoT load simulation.

**Fig. 11:**
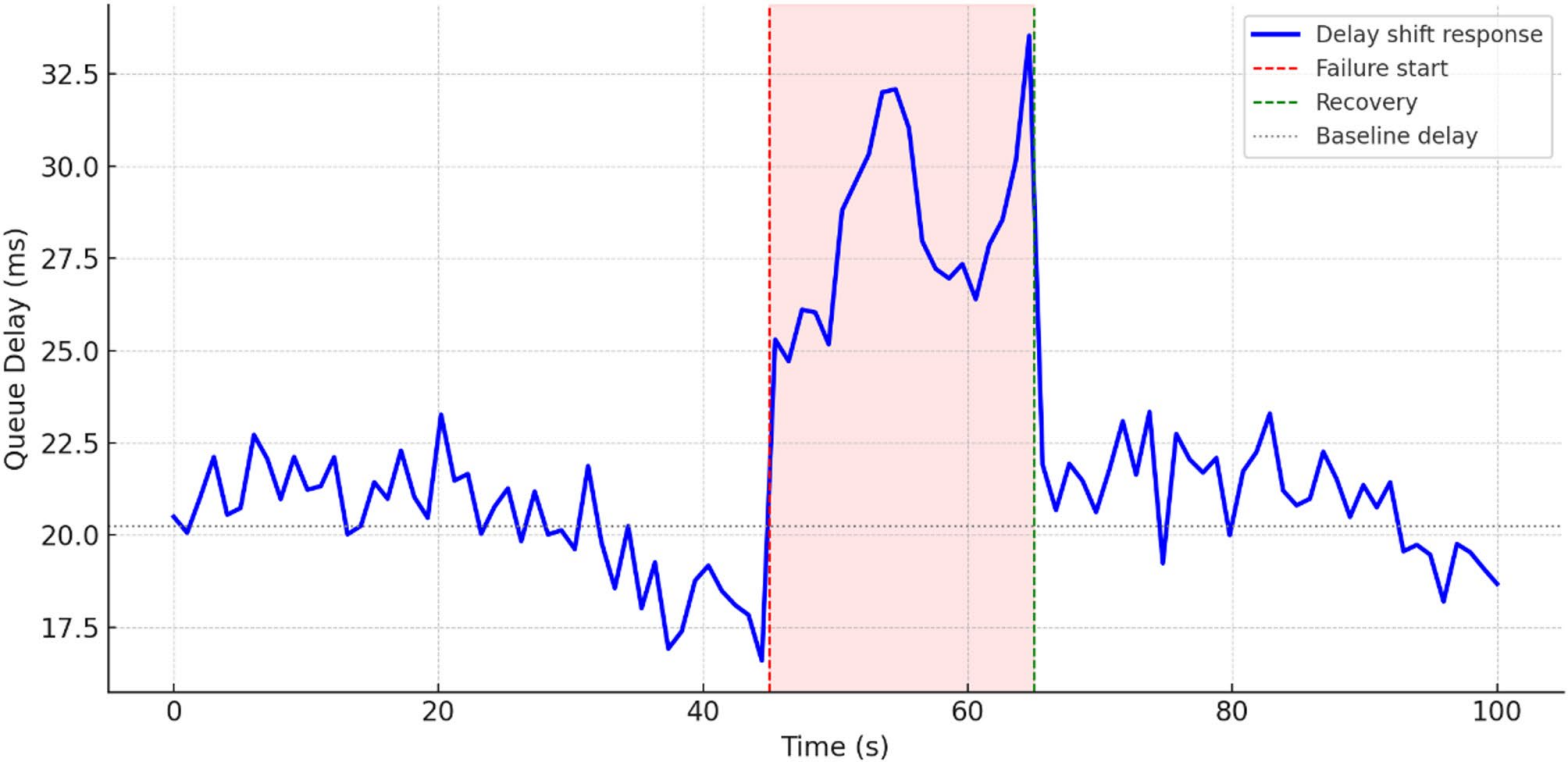
Delay shift response graph to an edge node failure.

Finally, within the modelling of emergency events, the response of the delay shift agent to an edge node failure was examined (a critical scenario for QoS in distributed IoT systems). The failure lasted within the interval [45; 65] s and simulated a partial loss of computational resources. Figure 11 presents the graph of processing delay dynamics (ms) over time (s). The red shaded area represents the failure period; vertical lines indicate the start and end of the disruption; the dashed grey line shows the baseline delay level under normal conditions.

During the failure, the delay shift exhibits a delay spike of 12–15 ms, followed by recovery to the baseline level after approximately 65 s with a lag of about 7 s. This dynamic fully aligns with the expectations based on expressions (14), (19), and (23), which describe the impact of node loss on average delay and the effectiveness of adaptive reallocation. Thus, the delay shift ensures not only efficient scheduling under typical scenarios but also demonstrates high resilience to adverse environmental changes.

The results presented in Sect. 3 systematically confirm the effectiveness of the delay shift agent under variable load and heterogeneous edge-IoT environments. In comparison with current analogues^22,24–26^ across four key metrics (mean response time, deadline violation ratio, latency std. dev, queue recovery time), delay shift demonstrated a consistent advantage. The mean response time was reduced by 12.6% compared to the SEE-MTS agent, while the latency standard deviation decreased by approximately 18%. The share of deadline violations was reduced by an average of 27%, and the average queue recovery time was shortened by 34%. The statistical significance of these results was confirmed by the Kruskal–Wallis test with *p* < 0.01. Accuracy analysis of delay shift approximations against real data (Orange D4D, Intel Lab) yielded MAPE ≤ 6.1%, indicating a close alignment with the formalised analytical models (8)–(10), which are based on input traffic intensity, buffer size, and processing speed. The behaviour of delay shift in the edge node failure scenario exhibited a controlled delay increase of 12–15 ms with adaptive recovery to the baseline level within 7 s following the incident. This stabilisation results from the adaptive task distribution logic formalised in expressions (14), (19), and (23), which account for changes in resource availability and the current state of queues. Therefore, delay shift demonstrated reliability, adaptability, and accuracy in both normal and extreme scenarios, confirming its potential for deployment in real edge-IoT ecosystems with QoS and fault-tolerance requirements.

## Conclusions

The rapid expansion of edge-IoT systems demands fundamentally new approaches to traffic service management under conditions of unstable load, limited resources, and hardware-induced phase delays. The relevance of this study lies in the fact that neglecting the internal temporal structure of request processing on peripheral devices leads to data loss, increased energy consumption, and degradation of service quality, particularly in mission-critical environments such as medical monitoring, automated manufacturing, or URLLC applications.

This study proposes a novel approach to intelligent delay management in edge-IoT environments by integrating an analytically controlled G/G/1 queuing system with a controllable service phase shift parameter (delay shift θ) and an adaptive selection policy trained via reinforcement learning. Unlike existing solutions, the model captures phase unavailability due to service initiation offsets, enhancing modelling precision under dynamic load conditions. For the first time, an analytical G/G/1 model with shifted service intensity is derived in the Laplace domain using a spectral method, enabling formal control of θ to ensure the stability and tunability of queuing-time characteristics. A new reward function is also introduced, tailored to the delay shift model, which simultaneously incorporates quality-of-service metrics (mean response time, deadline violation rate, queue recovery time) and device energy usage.

The proposed delay shift agent consistently outperformed all baseline methods with statistically significant differences (*p* < 0.01), as confirmed by post hoc analysis. According to Pareto analysis, it achieves an optimal trade-off between latency and energy consumption, confirming its suitability for deployment in energy-constrained edge-IoT environments with strict timing requirements.

The approach has practical applicability for developing adaptive request-handling systems in heterogeneous edge-IoT scenarios with variable traffic loads and energy constraints. The delay shift model enables local control over service initiation without centralized coordination, making it suitable for embedded systems with energy-efficient computing platforms – especially those running Embedded Linux. The reinforcement learning-based agent operates without prior knowledge of traffic statistics, making it applicable to dynamic contexts such as sensor networks, industrial edge computing, and smart infrastructure nodes. Empirical evaluation on three real-world datasets (Orange D4D, Intel Lab Sensor Data, and Edge Computing Dataset) demonstrated stable behaviour across a wide range of request rates (50–250 req/s), maintaining a well-balanced latency–energy trade-off.

Theoretical limitations of the model stem from the assumption of controllable delay shifting, i.e., programmable offsets in G/G/1 queues. This requires access to a queue scheduler capable of modifying service activation without violating service order, which may not be feasible on devices with rigid firmware or lacking such functionality. Moreover, the modelling assumes fixed arrival intensity within training episodes, reducing predictive accuracy under bursty traffic or heavy-tailed interarrival distributions. The spectral method in the Laplace domain requires numerical inversion under non-parametric service distributions, increasing computational overhead and limiting real-time applicability in systems with strict energy or latency budgets. Practical deployment also necessitates software-level access to scheduling policies, often unavailable on typical low-power edge platforms using RTOS or microcontrollers. In addition, the agent’s training demands multiple episodes, which can be impractical in high-risk environments like medical IoT or autonomous control.

Future research directions include extending the delay shift model to support heavy-tailed and highly variable traffic without requiring explicit distribution assumptions, thus enhancing its applicability to stochastic edge-IoT environments with bursty workloads^39^. Another avenue involves designing lightweight, energy-efficient agent variants using offline or transfer learning techniques, particularly for deployment on constrained edge platforms^40^. Further investigation will also explore the integration of delay-aware mechanisms with priority-based scheduling schemes and distributed multi-queue architectures, as required in clustered 5G infrastructures and smart manufacturing ecosystems^41,42^. Such enhancements aim to improve scalability, resilience, and responsiveness in mission-critical edge systems operating under extreme load conditions.

## Data Availability

The datasets used in this study are publicly available but subject to specific terms of use. The Intel Lab Sensor Data (Intel Berkeley Research Lab Sensor Data) is available via Kaggle: https://www.kaggle.com/datasets/divyansh22/intel-berkeley-research-lab-sensor-data. This dataset is provided under a CC0: Public Domain license, permitting unrestricted use. The Orange D4D dataset is accessible at the following repository: https://github.com/rmaestre/d4d-challenge. Access to this dataset is governed by the original Data Use Agreement as specified by the D4D Challenge organizers. The Edge Computing Dataset, used to complement the analysis, is openly available from the BUPT MEC Migration project repository: https://github.com/BuptMecMigration/Edge-Computing-Dataset. No additional data were generated during the current study.

## Abbreviations

ACM: Association for Computing Machinery
BA: Batch Arrival
BLE: Bluetooth Low Energy
CI: Confidence Interval
CPU: Central Processing Unit
DQN: Deep Q-Network
DRL: Deep Reinforcement Learning
GB: Gigabyte
IEEE: Institute of Electrical and Electronics Engineers
IoT: Internet if Things
KB: Kilobyte
MAC: Medium Access Control
MAPE: Mean Absolute Percentage Error
MDP: Markov Decision Process
NB: Number of Batches
QoS: Quality of Service
RAM: Random Access Memory
RED: Random Early Detection
RL: Reinforcement Learning
RT: Response Time
SEE-MTS: Self-Elastic Edge with Multi-Tier Scheduling
URLLC: Ultra-Reliable Low-Latency Communication

## List of Symbols

$\text{E}[\cdot]$: Expected value – average over all realisations of a random variable
$\text{P}(\cdot)$: Probability function – likelihood of an event or condition
$\text{Q}(\cdot)$: Queue length or state function (e.g., expected queue size or delay)
$\text{Mean}(\cdot)$: Arithmetic mean – average value (used in evaluation metrics)
$\text{Std}(\cdot)$: Standard deviation – quantifies variability in observed values
$\text{ReLU}(\cdot)$: Rectified Linear Unit – neural activation function
$\text{sofmax}(\cdot)$: Activation function for action selection probability distribution
$\text{g}(\cdot)$: Custom delay-shift function (used to regulate service delay, see eq. 19)
$\text{argmax}(\cdot)$: Argument of the maximum – used for selecting optimal actions in RL
$\text{Clip}(\cdot)$: Value clipping function – limits input to a fixed numerical range
$\text{r}(\cdot)$: Reward function – maps state-action pairs to scalar feedback in RL
$\triangle$: Delay shift parameter – applied to schedule or defer request execution†
$\sigma$: Standard deviation parameter in latency models
